# Effect of SiO*_x_* Nanocoating Deposition Route on Oxygen Evolution Reaction/Chlorine Evolution Reaction Selectivity of IrO*_x_* Anodes in Acidic Saline Water Electrolysis

**DOI:** 10.1002/cssc.71062

**Published:** 2026-09-20

**Authors:** Katherine Stephanie Encalada‐Flores, Koen de Graaf, Athina Tzavara‐Roussi, Bhavesh Chavan, Philipp Brüner, Thomas Grehl, J. Ruud van Ommen, Ruud Kortlever

**Affiliations:** ^1^ Process and Energy Department Faculty of Mechanical Engineering Delft University of Technology Delft Netherlands; ^2^ Department of Chemical Engineering Delft University of Technology Delft Netherlands; ^3^ IONTOF GmbH Münster Germany

**Keywords:** acidic saline water electrolysis, chlorine evolution reaction, fabrication–functionality relationship, oxygen evolution reaction, silicon oxide nanocoatings

## Abstract

Proton exchange membrane water electrolysis for green hydrogen production is typically designed for ultrapure water feeds. In offshore applications, for example, when coupled to desalination, residual chloride can still trigger the chlorine evolution reaction (CER), motivating the development of oxygen evolution reaction (OER)‐selective anodes. Silicon oxide (SiO_*x*_) nanocoatings exhibit chloride‐blocking behavior and suppress Cl_2_ formation. Previous studies used spin coating (SC) to fabricate SiO_*x*_ coatings, but enhanced OER selectivity was limited to smooth catalyst surfaces, and the coatings showed poor mechanical stability. Here, we introduce atomic layer deposition (ALD) as a vapor‐phase alternative and benchmark it against SC to investigate how the SiO_*x*_ fabrication method influences chlorine suppression on rough amorphous IrO_*x*_ anodes. SiO_*x*_‐coated anodes were evaluated in a low‐salinity acidic electrolyte using a rotating ring–disk electrode (RRDE) to quantify chlorine evolution and assess mechanical integrity. After 50 scans, plasma‐enhanced ALD and spray coating at a SiO_*x*_ thickness of 0.5 nm both reduced CER selectivity by approximately 36% relative to uncoated IrO_*x*_, whereas thermal ALD showed no selective CER suppression. These findings show that the chloride‐blocking behavior of ultrathin SiO_*x*_ nanocoatings depends strongly on the deposition route and likely arises from multiple coupled chemical, structural, and interfacial properties.

## Graphical abstract

How SiO_*x*_ nanocoatings are made matters. For acidic saline water electrolysis, silicon oxide (SiO_*x*_) layers prepared by different fabrication routes showed distinct abilities to selectively suppress the chlorine evolution reaction and enhance the oxygen evolution reaction selectivity. The results highlight coating chemistry, wettability, coverage, and mechanical stability as key descriptors linking the deposition route to chloride‐blocking performance.

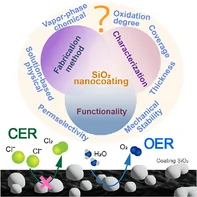

## 1 Introduction

Offshore water electrolysis powered by marine renewable energy sources offers a promising solution for producing cost‐competitive green hydrogen yet presents notable technological challenges [1, 2]. Integrating and co‐locating marine renewable technologies, such as floating offshore wind farms or photovoltaic systems, with e‐fuel technologies could enable renewable electricity to directly power hydrogen or ammonia production even in remote locations worldwide [3, 4]. In addition, selecting marine renewable energy locations with high energy potential and low water stress constraints such as the North Sea, coupled with the direct use of electricity with little or no transmission, could enable affordable green hydrogen production [5]. Multiple projects worldwide are combining marine renewable energy with electrolysis on fixed and floating platforms, with some already advancing to the pre‐commercial stage [1,6, 7, 8, 9, 10, 11]. To date, proton exchange membrane water electrolysis is the preferred electrolyzer technology for most offshore hydrogen production platforms because of its technological readiness, compatibility with dynamic power inputs, and compact design [4, 12]. This technology was developed based on a highly pure water feedstock; therefore, an upstream desalination process is required when seawater is used as the feedstock [13]. Many mature desalination technologies are already in use in the marine sector, with reverse osmosis (RO) being the preferred candidate for treating seawater [1, 4]. Single‐pass seawater RO significantly reduces dissolved salt concentrations, but the produced permeate typically requires further purification before use as electrolyzer feedwater [14]. Even trace concentrations of chloride ions are problematic, as their electro‐oxidation compromises cell efficiency and membrane stability, accelerates component degradation, and complicates product handling [13, 15, 16].

The kinetics and thermodynamics determine the competition between the targeted oxygen evolution reaction (OER) and the undesired oxidation of chloride ions at the anode during acidic saline water electrolysis [17]. The half‐cell reactions at the anode in acidic media are given in Equation (1) for the OER and Equation (2) for the chlorine evolution reaction (CER). The hydrogen evolution reaction takes place at the cathode [18, 19]. The CER is kinetically favored because it involves a two‐electron process, compared with the four‐electron OER, which proceeds through a more complex intermediate reaction mechanism [20].

(1) $$\begin{array}{l} 2H_{2}O⇌O_{2}\left(g\right)+4H^{+}+4e^{-}\\ E_{O_{2}/H_{2}O}^{0}=1.229\mathrm{Vvs}.\text{SHE} \end{array}$$

(2) $$\begin{array}{l} 2{\mathrm{Cl}}^{-}⇌{\mathrm{Cl}}_{2}\left(g\right)+2e^{‒}\\ E_{{\mathrm{Cl}}_{2}/{\mathrm{Cl}}^{-}}^{0}=1.3583\;V\;\mathrm{vs}.\text{SHE} \end{array}$$

In an acidic environment (pH < 2), thermodynamics favors the OER, as its standard equilibrium potential is lower than the CER ($E_{O_{2}/H_{2}O}^{0}<E_{{\mathrm{Cl}}^{‒}/{\mathrm{Cl}}_{2}}^{0}$). Nonetheless, this comparison alone does not reflect practical conditions, under which a significant overpotential (*η*) is required to drive the reactions. For instance, the OER requires a minimum overpotential of approximately 250 mV, thereby raising the onset potential to around 1.48 V versus reversible hydrogen electrode (RHE) [21]. On benchmark catalysts such as RuO_2_ and IrO_2_, the CER typically requires a lower overpotential than the OER to reach comparable current densities ($\eta_{\mathrm{OER}}>\eta_{\mathrm{CER}}$) [22]. Therefore, reaction competition is unavoidable, as there is no thermodynamically exclusive OER potential window under real experimental conditions. Several strategies have been explored to suppress chloride oxidation during the electrolysis of saline or low‐grade chlorine‐containing water [23, 24, 25, 26]. Among these strategies, encapsulation or membrane protection with ultrathin coatings (1–10 nm) has been shown to enhance electrocatalyst stability and tune reaction selectivity [27, 28, 29, 30, 31].

Ultrathin coatings have been reported to exhibit membrane functionality due to their selective transport properties, specifically, permselectivity, similar to those observed in membrane‐coated electrocatalysts [30, 31, 32]. Permselectivity is defined as the preferential permeation of specific ionic species through a membrane [33]. It is determined by several properties, including the density, free volume, porosity, flexibility, thickness, and hydrophilic properties, as well as the concentration and distribution of fixed charges within it [34, 35]. Matrix density and free volume determine the available transport pathways. In polymers, these pathways may change dynamically because of swelling in water, whereas they remain comparatively fixed in rigid mineral or inorganic ion exchangers [34]. Zhang et al. concluded that an optimal performance is a trade‐off between permeability and selectivity, where the concentration of fixed charged ions in the membrane (charge density) seems to have a greater impact than the thickness [35]. When fixed charges are present within the layer, oppositely charged counterions are electrostatically attracted to the membrane. This ion partitioning is governed by Donnan’s potential principle and contributes to electroneutrality at the membrane–solution interface [36]. In the case of ion exchange membranes, the homogeneous distribution of fixed charges across the bulk matrix allows the transport of a surplus charge of counter‐ions [37]. However, if the fixed charges are confined to the surface, the membrane may behave primarily as a charged barrier or electrostatic trap. The construction of Cl^−^ blocking membranes enables the development of OER‐selective anodes, allowing the use of chloride‐containing water streams as electrolyzer feedstock [30, 38, 39]. Silica (SiO_2_) coatings are a promising candidate for fabricating OER‐selective anodes due to their permselective behavior, which blocks Cl^−^ ions, and their chemical stability in acidic media [40, 41]. The Cl^−^ blocking functionality of the SiO_2_ coating is governed by multiple factors, including coating properties, electrolyte conditions, and the transport behavior of the reacting species. On the one hand, the (de)protonation of silanol groups (≡SiOH) determines the surface charge density and its binding capability with ions on a water‐immersed silica surface [42]. Silanol groups exhibit a Brønsted acid/base behavior (amphoteric surface), depending on the pH environment they are subjected to. The interfacial properties of amorphous silica are also strongly influenced by the concentration and distribution of surface silanol and siloxane groups, with silanols providing adsorption sites for water and other interacting species through hydrogen‐bonding and donor–acceptor interactions [43]. Because both the point of zero charge (PZC) and the isoelectric point of amorphous SiO_2_ in water are reported between pH 2 and 3, strongly acidic conditions below this range are expected to shift the amphoteric silanol equilibria toward protonated surface species, including positively charged (≡SiOH + H^+^ ↔ ≡SiOH_2_^+^) [44]. The protonation of surface silanol groups is a so‐called “activationless” process because it is fast and has an insignificant activation energy [45]. On the other hand, ion permeation is governed by three primary separation mechanisms: ion–membrane electrostatic interactions, pore size sieving, and hydration energy [34].

This work aims to understand how different fabrication methods influence the structural integrity, stability, and permselectivity of ultrathin SiO_*x*_ nanocoatings for OER‐selective anodes in acidic saline water electrolysis. To the best of our knowledge, previous SiO_*x*_ nanocoatings for this application have been fabricated only wet chemical deposition methods such as spin coating of polydimethylsiloxane (PDMS), followed by ozone oxidation. However, these coatings exhibited limited mechanical stability [40, 41]. Hence, we were motivated to use a chemical vapor deposition method, specifically atomic layer deposition (ALD). The principal attributes of ALD encompass uniformity and conformality, which are essential for achieving consistent thickness and composition through the deposited material [46, 47]. Additionally, we hypothesize that the chemical bonding between the catalyst and the ALD‐deposited nanocoating may improve the mechanical stability. First, we examine how the deposition method affects the electrochemical response, chemical composition, apparent double‐layer capacitance, $C_{\mathrm{dl},\mathrm{app}}$, wettability, and coverage of the nanocoating and its correlation with the relative change in molar selectivity toward OER and current response. Furthermore, the coated anodes were subjected to repeated cyclic voltammetry (CV), using a preestablished rotating ring–disk electrode (RRDE) quantification method, to assess their short‐term electrochemical and mechanical stability [48]. Later, the effect of membrane thickness was evaluated together with its mechanical stability. Overall, our findings highlight that, although the final material is nominally SiO_2_, the coating method can significantly impact its Cl^−^ blocking capability.

## 2 Experimental

### 2.1 Synthesis and Deposition of IrO*_x_* Catalysts on Glassy Carbon Substrates

IrO_*x*_ nanoparticles were deposited on mirror‐polished glassy carbon (GC) disks mounted in a Pine Research rotating disk electrode (RDE). The IrO_*x*_·nH_2_O colloid was synthesized following a procedure adapted from Vos et al. [30] by hydrolysis of Na_2_IrCl_6_·6H_2_O in 0.1 M NaOH at 90 °C, followed by acidification with HClO_4_ to pH ≈ 1 under cold stirring, yielding a dark blue dispersion. IrO_*x*_ was then electrophoretically deposited in a three‐electrode RDE configuration at 600 rpm by CA at 1.255 V versus Ag/AgCl to a total charge of 0.4 C after 10 conditioning CV scans. The resulting films were electrochemically preconditioned in Ar‐purged 0.5 M H_2_SO_4_ by CV until stable response. Full synthesis, deposition, and conditioning details are provided in Sections S1.1 and S1.2.

### 2.2 SiO*_X_* Nanocoating Deposition via Different Fabrication Methods

Silicon oxide (SiO_*x*_) nanocoatings were deposited on IrO_*x*_|GC anodes using three fabrication routes: thermal atomic layer deposition (T‐ALD), plasma‐enhanced atomic layer deposition (PE‐ALD), and spray coating (SC), followed by UV/ozone curing. T‐ALD was performed using SiCl_4_ and a water vapor/ozone co‐reactant, whereas PE‐ALD employed bis(diethylamino)silane (BDEAS) and O_2_ plasma, thus providing chemically distinct vapor‐phase deposition routes. SC served as a solution‐based control route using polydimethylsiloxane as the Si precursor. This approach enabled a direct comparison of how the three fabrication methods influence SiO_*x*_ chemistry, structure, and ion‐blocking functionality. Detailed operating parameters are provided in Section S1.3.

### 2.3 Chlorine Evolution Reaction and Oxygen Evolution Reaction Molar Selectivity Quantification

The OER versus CER selectivity was quantified using a RRDE setup via the method developed by Vos and Koper [48]. A low‐volume cell was filled with 12 mL of acid saline electrolyte (0.5 M H_2_SO_4_ + 30 mM NaCl) that had been degassed by bubbling argon gas for 5 min to ensure that dissolved oxygen would not affect OER quantification. The electrolyte was renewed for every electrode to ensure consistent Cl^−^ concentrations at the start of every experiment. RHE and Pt foil (placed parallel under the RRDE shaft) were used as reference electrode (RE) and counter electrode (CE), respectively. The Pt ring was electropolished, as described in Section S1.5. The quantification method consists of applying an oxidizing potential to the disk, where Cl^−^ ions and H_2_O are electrochemically oxidized into Cl_2_ (CER) and O_2_ (OER), respectively, while simultaneously maintaining the ring at a potential for the exclusive chlorine reduction reaction (CRR). This is done using a bipotentiostat technique, in which CV and chronoamperometry (CA) are run simultaneously. At the disk, CV was performed over a potential range from 1.1 to 1.55 V versus RHE at a scan rate of 10 mV s^−1^. Meanwhile, the ring potential was held at 0.95 V versus RHE to electrochemically reduce the chlorine generated at the disk into Cl^−^ ions. The ring current corresponds to the fraction of chlorine produced at the disk that reached the ring and is subsequently reduced ($i_{\mathrm{ring}}=i_{\mathrm{CRR}}$). Such a fraction is given by the collection factor (*N*) that was determined to be *N* = 0.237 for our system (further details are given in Section S1.5). By using Equations (3) and (4), the current for each competing reaction can be derived

(3) $$i_{\text{disk}}=i_{\mathrm{OER}}+i_{\mathrm{CER}}$$

(4) $$i_{\mathrm{CER}}=\frac{\left|i_{\text{ring}}\right|}{N}$$

Then, the molar selectivity toward CER can be obtained through Equation (5) and is denoted by the symbol *S*_CER_. In the present study, the current response values, $i_{\mathrm{CER}}$ and $i_{\mathrm{OER}}$, were measured at a disk potential of 1.55 V versus RHE. This potential is high enough for both reactions to occur and compete, but not so high that bubble formation disturbs the measurements in the RRDE setup. Assuming that the total disk current (or total moles of electrons) arises exclusively from the CER or OER, the molar selectivity toward OER $\left(S_{\mathrm{OER}}\right)$ can be inferred by Equation (6). This expression is derived by converting the partial currents associated with CER and OER into molar production rates via Faraday’s law while accounting for the different numbers of electrons transferred in each reaction. Molar selectivity can be expressed as a fraction or as a percentage.

(5) $$S_{\mathrm{CER}}=\frac{\frac{i_{\mathrm{CER}}}{2}}{\frac{i_{\mathrm{CER}}}{2}+\frac{i_{\mathrm{OER}}}{4}}$$

(6) $$S_{\mathrm{OER}}=1‐S_{\mathrm{CER}}$$

By comparing the change of molar selectivity after the coating $\left(S_{\text{coated}}\right)$ relative to its initial value $(S_{\text{uncoated}}),$ it is possible to quantify the relative percentage change in molar selectivity (ΔS) as shown in Equation (7). Similarly, Equation (8) can be used to monitor the relative percentage change in current (Δi(%)). Relative changes are computed with respect to each particular electrode’s uncoated baseline, either S or i. This is because current baselines may vary slightly across electrodes due to differences in the IrO_*x*_ catalyst loading between batches and experiments. Therefore, quantitative comparison is based on the magnitude of change (|Δ*X*|), and the sign indicates whether the change is a decrease (negative value) or an increase (positive value).

(7) $$\Delta S=\left(\frac{S_{\text{coated}}-S_{\text{uncoated}}}{S_{\text{uncoated}}}\right)\cdot 100\%$$

(8) $$\Delta i=\left(\frac{i_{\text{coated}}-i_{\text{uncoated}}}{i_{\text{uncoated}}}\right)\cdot 100\%$$

The $S_{\text{uncoated}}$ value was calculated for the 10^th^ CV scan, at which no further changes in the current signal were observed in either the disk or the ring electrodes after a slight current increase that occurred within the first five CV scans. On the other hand, the $S_{\mathrm{CER},\text{coated}}$ value was calculated for the 50^th^ CV scan as a short‐term durability test, evaluating the coated anode’s ability to maintain electrochemical performance over electrochemical cycling.

As previously reported in the literature, bubble formation at the Teflon spacer between the disk and ring electrode interfered with the accuracy of the ring current measurement by yielding a lower value than reality [49]. Consequently, a manual bubble removal procedure was incorporated every ten CV scans. It consists of temporarily pausing the experiment, both from the potentiostat software and the rotator, during the reverse scan at potentials near 1.0 V versus RHE. The adhered bubbles were gently removed using argon gas flow, and the experiment was resumed only after the system had returned to its original operating conditions. Generally, bubble formation at the Teflon spacer of uncoated electrodes was significantly more pronounced than that of coated ones. This is expected considering the decrease in current response upon coating, as discussed in Section 3.2.

### 2.4 Anode Characterization

The surface composition and oxidation states were analyzed by X‐ray photoelectron spectroscopy (XPS, Thermo Fisher Scientific, monochromatic Al Kα source) with spectra referenced to C‍ 1s = 284.8 eV. Coating coverage and the ALD growth mechanism were examined using low‐energy ion scattering (LEIS, Qtac 100, IONTOF) employing ^4^He^+^ and ^20^Ne^+^ ions. The apparent double‐layer capacitance, $C_{\mathrm{dl},\mathrm{app}}$ was determined by CV in an Ar‐purged 0.5 M H_2_SO_4_ electrolyte before and after coating. The apparent water contact angle was measured in air by the sessile drop method using an OCA 25 contact angle instrument (DataPhysics Instruments GmbH). Morphological inspection and thickness calibration were conducted by scanning electron microscopy (SEM) (JEOL IT800SHL). Thickness was determined with spectroscopic ellipsometry (J.A. Woollam alpha‐SE) on Si witness wafers. Detailed instrumental parameters and fitting procedures are provided in Section S1.5.

## 3 Results and Discussion

### 3.1 Rotating Ring–Disk Electrode Response Showing Coating Molar Selectivity Loss Over Cycling

**FIGURE 1:**
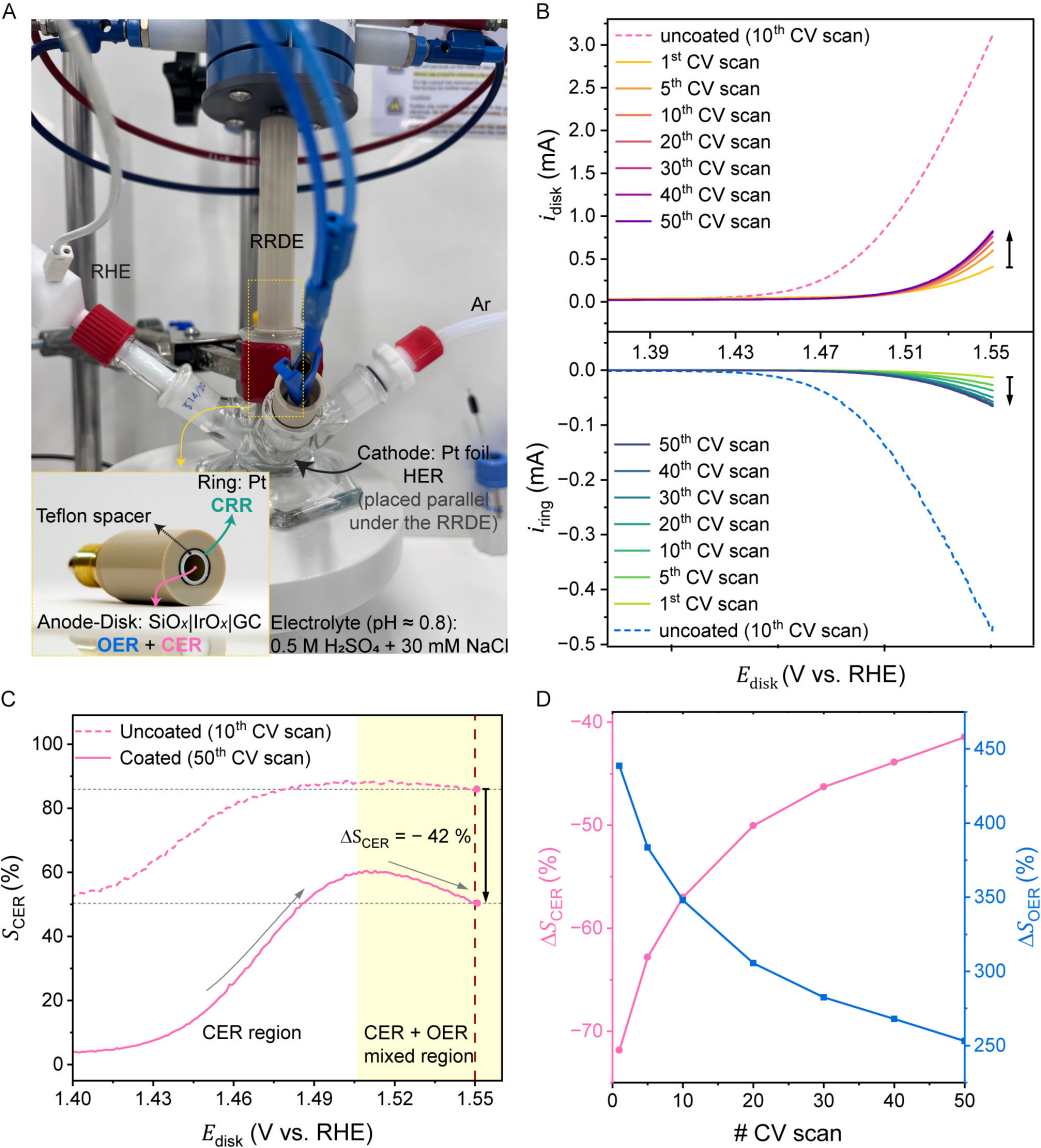
(A) Photograph of the experimental RRDE setup. The following panels show representative electrochemical plots for an SC‐fabricated anode coated with a nominal SiO_*x*_ layer of 0.5 nm. (B) Ring and disk current responses before and after nanocoating, as a function of increasing numbers of CV scans. (C) Molar selectivity toward the CER (*S*_CER_) versus disk potential for the last evaluated electrochemical state before and after the SiO_*x*_ coating. A down‐pointing arrow represents the relative percentage change in molar selectivity as a decrement. (D) Relative percentage change in molar selectivity for the CER and the OER versus the number of CV scans. In all cases, only the forward scan of each CV is shown.

Figure 1A shows a photograph of the experimental RRDE setup configuration, including the reactions taking place at the disk and ring. Figure 1B presents the disk and ring currents as a function of the disk potential for a spray‐coated electrode with a nominal SiO_*x*_ layer thickness of approximately 0.5 nm, tested following the electrochemical protocol detailed in Section 2.3. The top panel depicts the total disk current from the combined OER and CER reactions, while the bottom panel shows the current from the CRR. In both panels, the currents corresponding to the coated anode increase in magnitude upon repetitive CV, indicating a decline in OER molar selectivity. The most significant current increase occurs until the 30^th^ CV scan, after which the increase is less pronounced. Figure 1B graphically supports the selection of the 50^th^ CV scan for the analysis of coated electrodes, as described in Section 2.3. It is worth noting that despite the coating’s performance decay, the $i_{\text{ring},\text{coated}}$ after 50 CV scans remains significantly lower than the $i_{\text{ring},\text{uncoated}}$, showing that the nanocoating still effectively suppresses the CER. After processing the data plotted in Figure 1B through Equation (5), it is possible to calculate the molar selectivity versus disk potential, as shown in Figure 1C.

Figure 1C shows the molar selectivity toward CER ($S_{\mathrm{CER}}$) as a function of disk potential for the electrode before and after coating. In the CER‐dominated region, $S_{\mathrm{CER}}$ increases with disk potential, reaching a maximum at  ~1.5 V versus RHE. This rise in the CER molar selectivity is interrupted once the OER initiates, leading to a mixed CER + OER regime in which the CER molar selectivity decreases. The permselective properties of the coating inhibit chloride ion permeation into the catalyst, as evidenced by higher CER molar selectivity values for the uncoated electrode compared to the coated electrode. Notably, the analysis of the $S_{\mathrm{CER}}$ value is only electrochemically significant above 1.42 V versus RHE (the theoretical onset of CER [50]). Therefore, the $S_{\mathrm{CER}}$, for coated and uncoated electrodes, was evaluated at 1.55 V versus RHE. This potential, which is higher than the practical onset potentials for OER and CER, allows both reactions to be significantly active and competing without excessive bubble formation that would disturb the RRDE measurements. Finally, in Figure 1D we evaluate the relative percentage change in molar selectivity (ΔS) for the CER and the OER upon an increasing number of CV scans. Across 50 CV scans, the relative percentage reduction in CER molar selectivity decreased by 22 percentage points, from 78% at scan 1 to 56% at scan 50, suggesting a gradual weakening of the chloride‐blocking capability of the nanocoating.

### 3.2 Deposition‐Dependent Oxygen Evolution Reaction/Chlorine Evolution Reaction Selectivity: Coupled Effects of Coating Chemistry, Coverage, and Interfacial Properties

**FIGURE 2:**
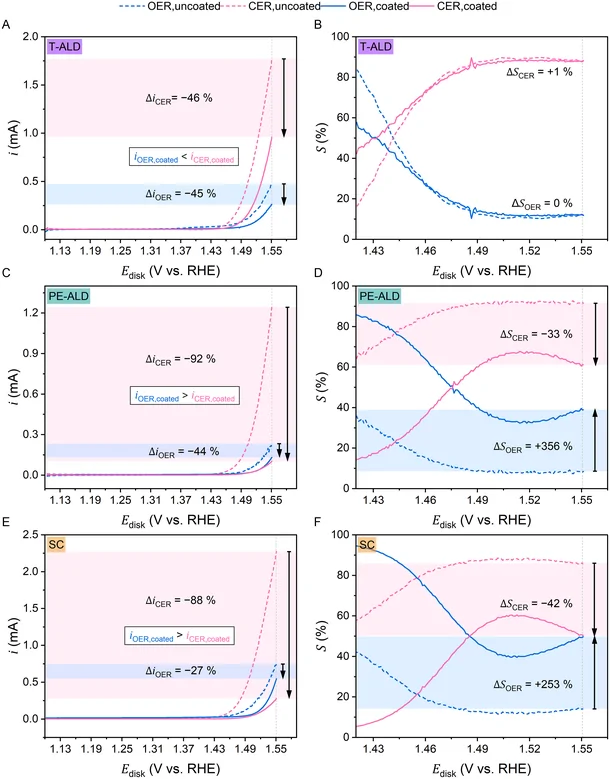
Linear sweep voltammogram corresponding to the forward scan of CV (left column) and its corresponding molar selectivity versus disk potential plot (right column) for a representative electrode before and after SiO_*x*_ coating fabricated via (A,B) T‐ALD, (C,D) PE‐ALD, and (E,F) SC methods. In all cases, the nominal thickness of the SiO_*x*_ layer is approximately 0.5 nm. The depicted polarization curves correspond to the last CV forward scan obtained from the RRDE in each electrode state (the 10th CV scan for the uncoated state and the 50th CV scan for the coated state) as detailed in the methodology. Correspondingly, the molar selectivity is reported as a percentage for readability, and the shaded area corresponds to the relative percentage change in current (Δi) or molar selectivity (ΔS) with respect to the same electrode subject before coating.

Figure 2 compares the performance of representative coated anodes fabricated via T‐ALD (A,B), PE‐ALD (C,D), and SC (E,F) coating methods. In all cases, the nominal thickness of the SiO_*x*_ nanocoating is approximately 0.5 nm. For the electrochemical results, Figure 2 (left column) shows that at 1.55 V versus RHE, regardless of the coating method, the currents for both competing reactions (CER and OER) are reduced (down‐pointing arrow) after applying an ultrathin SiO_*x*_ nanocoating compared to those obtained without the coating. Also, a shaded area graphically illustrates the relative percentage reduction in current (Δi) for each reaction. A noticeable difference is observed for the T‐ALD electrode (Figure 2A) where the magnitude of the relative percentage reduction in current is comparable for both competing reactions ($\left|\text{Δ}i_{\mathrm{CER},T.\mathrm{ALD}}|\approx |\text{Δ}i_{\mathrm{OER},T.\mathrm{ALD}}\right|$). Conversely, in the PE‐ALD and SC methods (Figure 2C,E) the relative percentage reduction in CER current is significantly larger in magnitude than that in the OER ($\left|\text{Δ}i_{\mathrm{CER}}|>|\text{Δ}i_{\mathrm{OER}}\right|$), indicating preferential CER suppression. For the PE‐ALD and SC coating methods, uncoated electrodes exhibit higher currents for the CER than the OER $(i_{\mathrm{OER},\text{uncoated}}<i_{\mathrm{CER},\text{uncoated}})$, as the CER is thermodynamically and kinetically more favorable than the OER under practical operational conditions. After coating, this trend is reversed, favoring the OER $\left(i_{\mathrm{OER},\text{coated}}>i_{\mathrm{CER},\text{coated}}\right)$ with the OER current curve lying above the CER one. Correspondingly, the coated electrodes exhibit higher onset potentials for both the CER and OER compared with the uncoated electrodes, with the shift being more pronounced for the CER. Consequently, the nanocoating reverses the typical onset potential order, with the CER now initiating at higher potentials than the OER, evidencing improved OER molar selectivity compared with the uncoated electrodes. This is in line with the literature, which reports an effective increase in CER overpotential attributed to the depletion of Cl^−^ ions near the buried catalyst interface due to an increase in the diffusion layer thickness [30]. However, higher onset potentials of both reactions after coating imply that the cell requires larger overpotentials to initiate the reactions, thereby increasing the energy input required.

Moreover, molar selectivity trends were found to be dependent on the deposition method. When comparing at 1.55 V versus RHE, Figure 2B shows that the T‐ALD SiO_*x*_ coating yields to effectively zero relative change in CER or OER molar selectivities. In contrast, PE‐ALD Figure 2D) and SC (Figure 2F) coatings exhibit a decrease in the relative percentage change in CER molar selectivity $\left(\text{Δ}S_{\mathrm{CER}}<0\right)$ coupled with an increase in OER molar selectivity $\left(\text{Δ}S_{\mathrm{OER}}>0\right)$. In Figure 2D,F, equal absolute changes for CER and OER (blue‐ and pink‐shaded areas) translate into a modest relative percentage decrease for CER but a significant relative percentage increase for OER. This is because, at a low baseline molar selectivity, any change has a much greater impact than at a higher molar selectivity starting point. Vos et al. reported essentially no CER suppression ($\text{Δ}S_{\mathrm{CER}}\approx ‐4\%$) with spin‐coated + UV‐cured SiO_*x*_ coating of 5–20 nm under similar conditions [41]. In contrast, our 0.5 nm coatings, coated via PE‐ALD or spray coating on comparable anodes, exhibit markedly higher molar selectivity. This comparison indicates that the deposition method, rather than nominal thickness alone, strongly influences CER suppression.

To understand the coating method‐dependent suppression of the CER, we characterized the chemical composition and coverage of the SiO_*x*_ films fabricated via different methods. Ex situ ellipsometry mapped thickness versus ALD cycle number for ALD methods or versus precursor concentration for SC to guide a nominal thickness baseline comparison (Figure S6). Throughout this work, thickness values are reported as “nominal thicknesses” measured on simultaneously coated Si|SiO_*x*_ witness wafers. While these values provide a reliable measure of the deposited material under identical conditions, the actual local thickness on the nanostructured IrO_*x*_ catalyst surface may differ due to its rough and porous morphology (elaborated in Section S1.5).

**FIGURE 3:**
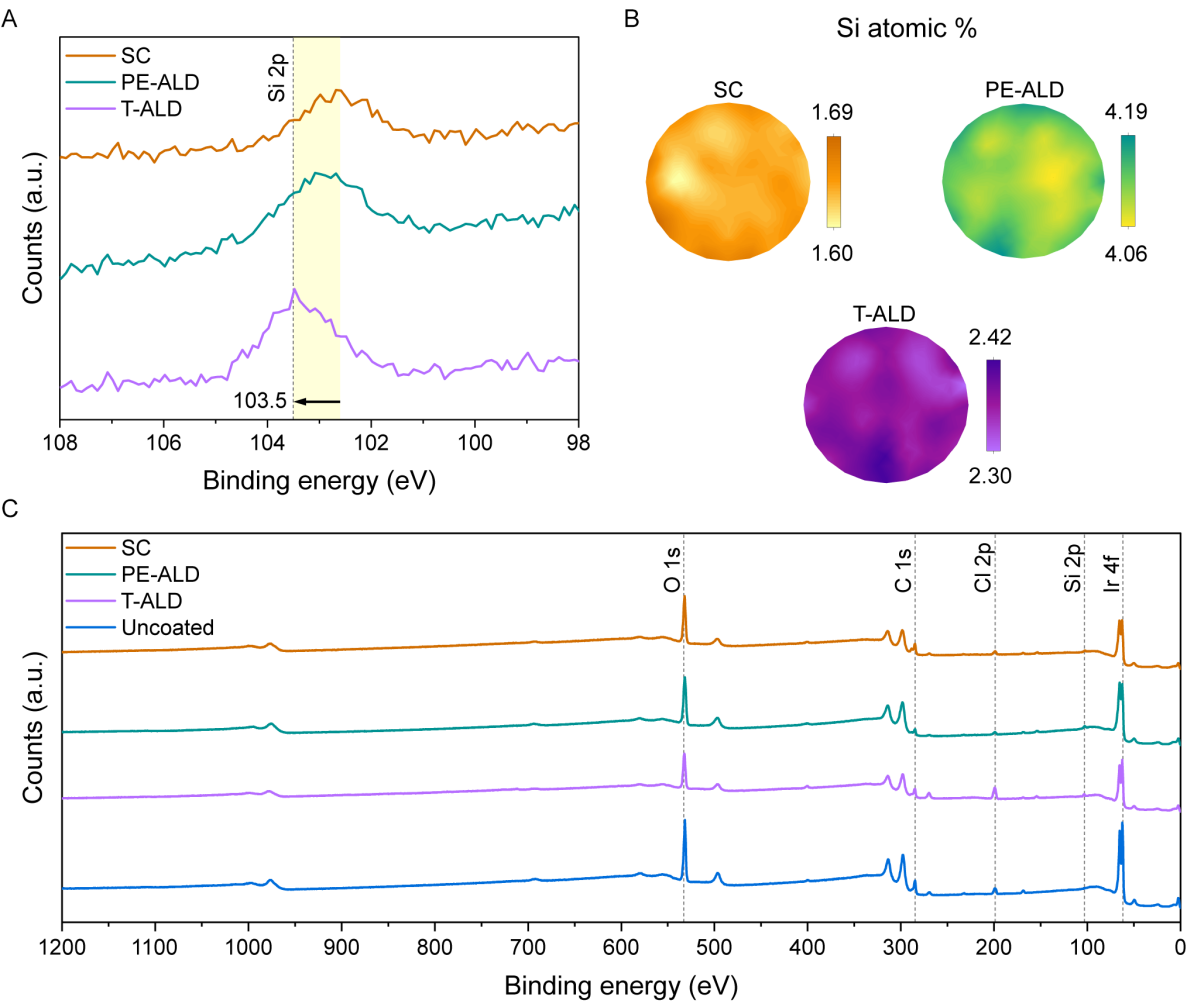
(A) Comparative XPS high‐resolution Si 2p spectra of coated anodes (GC|IrO_*x*_|SiO_*x*_) prepared via different methods, measured before electrochemical testing. (B) Si atomic percentage contour plot constructed out of 49 points radially distributed in a circular area of the fabricated anodes. The studied area corresponded to a center circle of a diameter of 4.5 mm. (C) Comparative XPS spectra survey of SiO_*x*_‐coated electrodes (GC|IrO_*x*_|SiO_*x*_) fabricated with different coating methods versus an uncoated electrode (IrO_*x*_|GC). In the case of the ALD coatings, the electrodes correspond to a five‐cycle, whereas in the SC case, the concentration of the solution used was 10 mg mL^−1^. All spectra were referenced to the adventitious C 1s peak at 284.8 eV.

High‐resolution XPS spectra shown in Figure 3A (108–98 eV) display a Si 2p feature characteristic of SiO_2_, confirming successful deposition for all coating methods while revealing method‐dependent variations in silica oxidation states. The high‐resolution Si 2p peak for the coated anodes, expected at approximately 103.5–103.6 eV for stoichiometric SiO_2,_ appears slightly shifted toward a lower binding energy, consistent with silicon suboxides [51, 52, 53]. The largest shift, of ~1 eV, is observed with the SC electrode, followed by the PE‐ALD electrode. In contrast, the T‐ALD electrode exhibited no significant peak shift. Comparable lower‐binding‐energy shifts have also been observed in UV/ozone‐converted PDMS‐derived SiO_*x*_ films and in PE‐ALD SiO_*x*_ coatings, where they were associated with suboxide species and oxygen‐deficient silicon environments [54, 55]. Thus, the shift observed here is more consistent with substoichiometric SiO_*x*_ than with fully oxidized SiO_2_. The presence of silicon suboxides Si^*x*+^ (*x* = 1, 2, or 3) arises from Si atoms that remain oxygen undercoordinated. Si reaches its maximal electron deficiency (highly oxidized state *x* = 4) when Si is entirely surrounded by oxygen atoms [51]. Due to the low Si 2p signal intensity of the ultrathin and partly discontinuous SiO_*x*_ coatings, deconvolution of the Si 2p envelope into individual silicon oxidation‐state components was not considered sufficiently robust. The number of constrained components required to represent the possible suboxide states would be large relative to the available signal‐to‐noise ratio. The O 1s envelope was likewise not used as a quantitative measure of silanol density because it contains overlapping contributions from IrO_*x*_ lattice oxygen, Ir─OH, Si─O─Si, Si─OH, adsorbed water, and possible residual electrolyte species. Moreover, ex situ XPS cannot independently establish that hydroxylated sites were protonated under the electrochemical operating conditions. Accordingly, the present XPS results support deposition‐dependent differences in the SiO_*x*_ chemical state but do not directly quantify silanol density, surface charge, or silanol protonation.

Figure 3B shows a relatively homogeneous distribution of Si atomic percentages across the analyzed area, irrespective of the coating technique. The absolute variation (max–min), normalized by the mean of the range, is below 6% across the analyzed surface for all fabrication methods. In addition, no chemical contamination is measured as a result of the different coating procedures, as shown by the XPS survey obtained after the SiO_*x*_ deposition (Figure 3C). Peaks associated with O, C, Cl, and Ir are present in all electrodes, including the blank. The uncoated IrO_*x*_|GC electrode exhibits a Cl 2p_3/2_ feature near 199 eV, which is attributed to residual [IrCl_6_]^2−^ coming from the IrO_*x*_ nanoparticle synthesis. For further confirmation, an XPS survey was conducted on a control substrate (Si wafer) for the T‐ALD fabrication method, which forms HCl as a by‐product during SiO_2_ synthesis. No Cl signal was detected on the coated Si control (Figure S10), confirming that this peak is exclusive to the catalyst layer and not originating from unreacted precursor or side reactions during nanocoating deposition.

To assess the chemical stability of the nanocoating after operation, post‐cycling XPS measurements were performed on nominal 0.5 nm SiO_*x*_‐coated electrodes, as shown in Figure S11. The Si 2p signal remained at a binding energy comparable to that observed before electrochemical testing, with no significant shift or appearance of additional Si species after cycling. These observations suggest that the remaining SiO_*x*_ preserves a similar chemical environment during operation and provide no evidence for substantial changes in the Si oxidation state.

**FIGURE 4:**
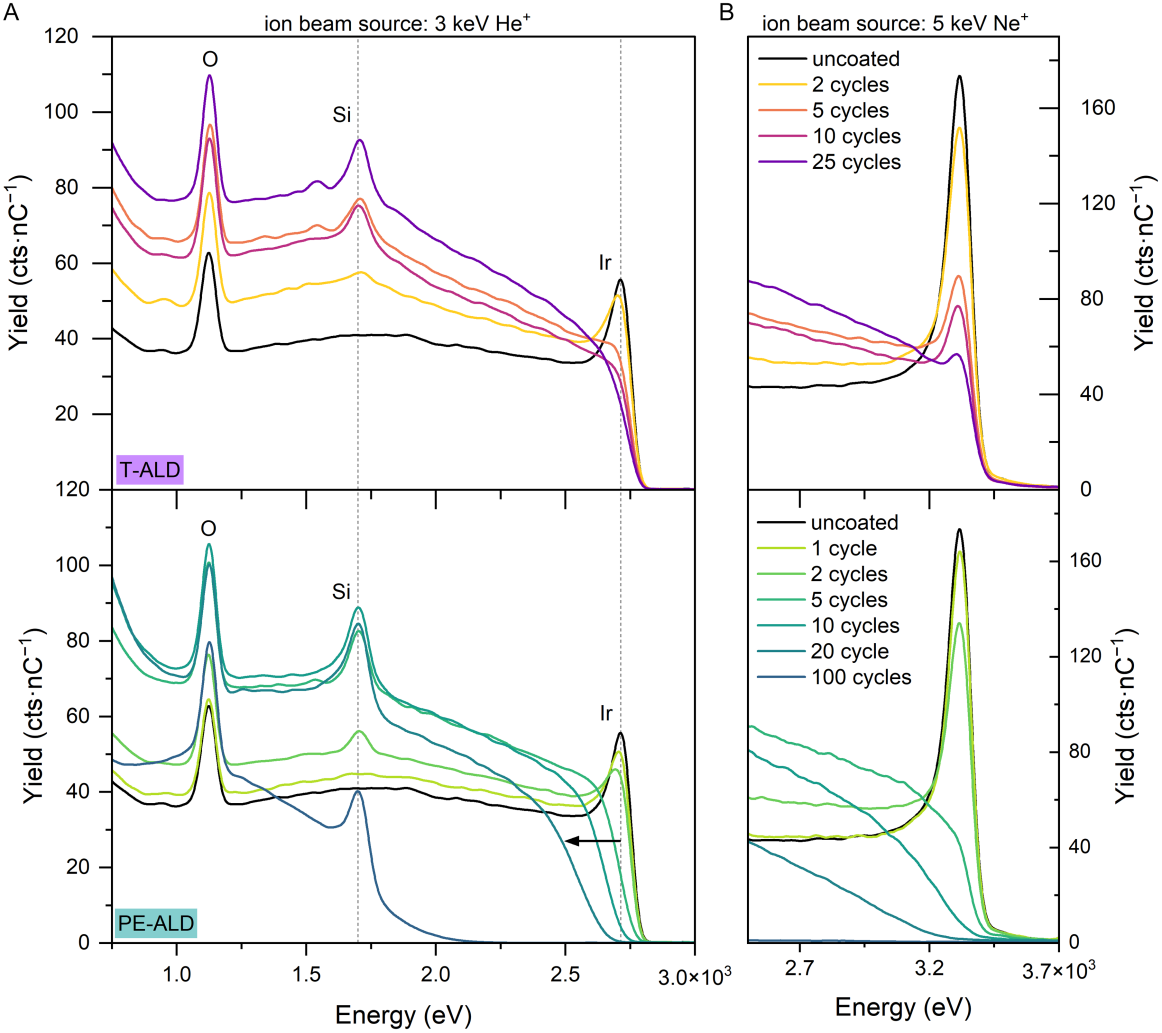
LEIS spectra obtained using two ion beam sources: (A) 3 keV ^4^He^+^ and (B) 5 keV ^20^Ne^+^, for SiO_*x*_ coatings fabricated by (top) T‐ALD and (bottom) PE‐ALD. The spectra are used to analyze the SiO_*x*_ coating coverage of the buried IrO_*x*_ catalyst, for which peaks corresponding to O, Si, and Ir are depicted.

LEIS analysis confirmed that the SiO_*x*_ films remained discontinuous within the range of ALD cycles investigated in this work while also providing information on the ALD growth mechanism. Figure 4A shows that as the number of ALD cycles increases, the Ir peak shifts toward lower binding energies and its intensity decreases. This is accompanied by a simultaneous increase in the Si peak intensity. The simultaneous detection of Si and exposed Ir during the early stages of both ALD variants confirms a Volmer–Weber growth mechanism, also known as island growth [56]. In Figure 4B (bottom panel), the PE‐ALD SiO_2_ film becomes continuous after 20 cycles, evidenced by the Ir peak’s shift to lower energy and subsequent disappearance. The film’s full closure is not achieved for the T‐ALD, even at a slightly higher number of cycles than the PE‐ALD, namely 25 (Figure 4B, top panel). Thus, we conclude that the nucleation density for the PE‐ALD is higher than that of T‐ALD, which is attributed to the higher reactivity of plasma‐generated species that create more active sites for nucleation [57]. This finding is particularly relevant, as it demonstrates that a fully closed SiO_*x*_ nanocoating is not required to achieve OER selectivity. Previous studies suggested that a defective/incomplete nanocoating or the rough catalyst morphology was responsible for the lack of OER selectivity [41]. In contrast, our results demonstrate that significant OER molar selectivity can still be achieved even with a noncontinuous nanocoating on an equivalent rough IrO_*x*_ catalyst. In addition, we show that discontinuity alone does not account for their different electrochemical behavior. Although both ALD coating routes exhibit island‐like growth at low cycle numbers, only the PE‐ALD route suppressed CER selectivity. Coating classified as noncontinuous can still differ substantially in island size and density, spacing, and exposed catalyst area. This leads to differences in the formed interisland gaps, which influence ion and molecular transport. Differences in the SiO_*x*_ islands may alter pathways by partially obstructing the catalyst surface, changing local tortuosity, and modifying the hydration and ionic environment near accessible IrO_*x*_ regions. Consistent with the XPS analysis, the LEIS evidence of discontinuous coverage is consistent with the observation of SiO_*x*_ suboxides presence (Si 2p shift), while not excluding an incomplete oxidation contribution.

To further examine how the deposition route modifies the coating/electrolyte interface, apparent double‐layer capacitance and water contact angle measurements were performed. The complete capacitance analysis is shown in Table S3 and Figures S12 and S13, while the contact angle results are shown in Figure S14. At a nominal coating thickness of approximately 0.5 nm, $C_{\mathrm{dl},\mathrm{app}}$ increased from 22.42 to 30.87 µF after PE‐ALD deposition and from 19.73 to 22.77 µF after SC, corresponding to relative increases of 37.7% and 15.4%, respectively. In contrast, T‐ALD decreased $C_{\mathrm{dl},\mathrm{app}}$ from 25.55 to 14.22 µF, corresponding to a relative decrease of 44.3%. Consistent with this trend, PE‐ALD and SC decreased the apparent water contact angle relative to uncoated IrO_*x*_, whereas T‐ALD produced a pronounced increase. Thus, preferential CER suppression coincided with greater apparent wettability and interfacial charge storage response for PE‐ALD and SC, while T‐ALD produced the least wettable surface, decreased $C_{\mathrm{dl}}$, and caused no appreciable change in OER/CER molar selectivity. The lower contact angles of PE‐ALD and SC correlate with their more substoichiometric SiO_*x*_ chemical environments hypothesis and may reflect deposition‐dependent differences in the nature and accessibility of surface hydroxyl and siloxane groups. Surface OH groups have been associated with enhanced silica wettability [58], as silanols provide hydrogen‐bonding sites for water, whereas siloxane‐rich regions exhibit a more hydrophobic character [43]. Surface ionization and interfacial energetics may also influence oxide wettability beyond the nominal position of the solution pH relative to the PZC [59]. Although the relative contributions of silanol and siloxane groups were not measured directly, the results demonstrate that the deposition route strongly influences SiO_*x*_ wettability and interfacial charge storage behavior.

**FIGURE 5:**
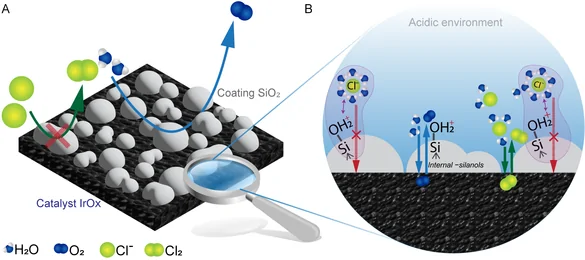
Schematic representation of (A) noncontinuous PE‐ALD‐fabricated SiO_*x*_ coating the catalyst (IrO_*x*_|GC). (B) Possible transport pathways through the discontinuous SiO_*x*_ nanocoating. Chloride‐blocking behavior may involve coupled electrostatic interactions, partial ion dehydration, steric constraints, and transport through the SiO_*x*_ matrix and interisland gaps.

Another possible contribution to CER suppression is the electrostatic interaction of Cl^−^ with protonated silanol sites, as schematically shown in Figure 5. Silanol‐terminating groups may originate as intrinsic surface species during ALD growth, incomplete condensation in UV/ozone‐oxidized PDMS‐derived coatings, or rehydroxylation of dehydroxylated silica upon water exposure [60, 61, 62]. Under our experimental conditions (pH = 0.8), the surface silanol groups can undergo protonation, generating positively charged sites capable of interacting with anions. The interaction with Cl^−^ could alter the interfacial distribution, residence time, or effective mobility of chloride within the coating and near the buried catalyst interface. In addition, for the discontinuous coatings studied here, transport may proceed through interisland gaps and SiO_*x*_|IrO_*x*_ boundary regions. Interisland gaps are not equivalent to defined nanopores, but both can provide confined transport environments in which surface chemistry influences. Molecular dynamics simulations and experimental diffusion measurements indicate that silica surface chemistry and confinement affect ion distribution and mobility within aqueous electrolytes in silica nanopores [63, 64]. Simulations specifically predict Cl^−^ accumulation near highly protonated surfaces [64]. Partial dehydration may further influence ion transport through confined pathways [40], whereas the transport of neutral O_2_ and Cl_2_ may depend on the relationship between the molecular size and the dimensions of the available pathways.

### 3.3 Current Loss and Oxygen Evolution Reaction Selectivity

**FIGURE 6:**
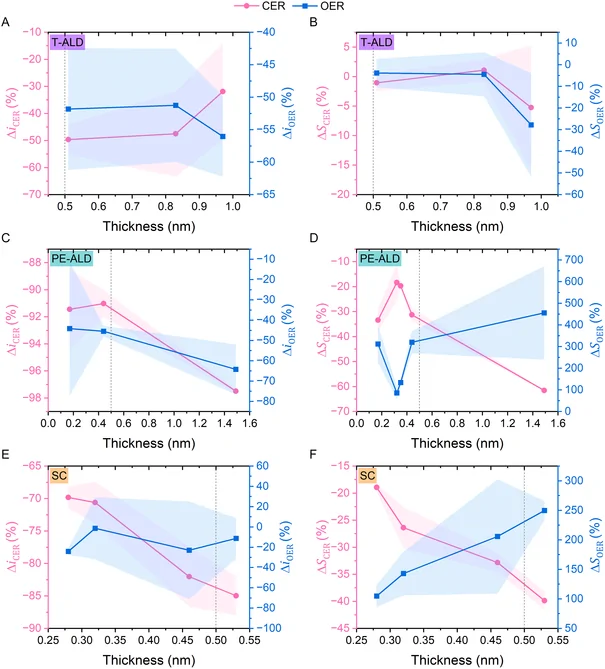
Relative percentage change in molar selectivity (ΔS) and current (Δi) toward CER (pink) and OER (blue), calculated using Equations (7) and (8), for increasing thicknesses of SiO_*x*_ coatings made with (A,B) T‐ALD, (C,D) PE‐ALD**,** and (E,F) SC. The molar selectivity of coated and uncoated electrodes was measured at 1.55 V versus RHE. The nominal thickness of each nanocoating was measured by spectroscopic ellipsometry, as shown in Figure S6, where a vertical reference dashed line at 0.5 nm is drawn for comparison purposes between the fabrication techniques that exhibited permselectivity, PE‐ALD and SC. Each curve is shown with a shaded error band representing the standard deviation of at least two replicas per sample.

In this section, we evaluate how the relative percentage change in current and molar selectivity varies with the thickness of the SiO_*x*_ nanocoating. Figure 6 compiles the Δi, and ΔS values obtained from Equations (7) and (8) for various nanocoating thicknesses. The relative percentage reduction follows a general descending trend for both competing reactions. At a comparable nominal SiO_*x*_ nanocoating thickness of 0.5 nm, as a value of comparison, the relative percentage reduction in CER current is comparable in magnitude for the PE‐ALD ($\text{Δ}i_{\mathrm{CER},\mathrm{PE}\cdot \mathrm{ALD}}\approx \text{−}91\%$) and the SC ($\text{Δ}i_{\mathrm{CER},\mathrm{SC}}\approx \text{−}84\%$) (Figure 6C,E). However, the relative percentage reduction in OER current for the PE‐ALD ($\text{Δ}i_{\mathrm{OER},\mathrm{PE}\cdot \mathrm{ALD}}\approx \text{−}46\%$) is larger in magnitude than its counterpart for the SC ($\text{Δ}i_{\mathrm{OER},\mathrm{SC}}\approx \text{−}18\%$). The larger OER current loss observed for the PE‐ALD nanocoatings may reflect stronger coating/catalyst interfacial bonding and/or more obstruction of accessible IrO_*x*_ active sites than in the spray‐coated film. In the case of the T‐ALD (Figure 6A), the relative current reduction for OER and CER remains approximately constant across all tested thicknesses, with values around 50%, though with significant error bars. This is the result of having a mixture of positive and negative values for the $\text{Δ}i_{\mathrm{CER}}$ replicates of the same coating thickness. Although this behavior may appear counterintuitive, when the magnitude of the anode’s total current reduces, the reallocation of current for the CER and the OER can yield to $i_{\mathrm{CER},\text{coated}}>i_{\mathrm{OER},\text{coated}}$ even for the case of favorable OER molar selectivity. In general, increasing the SiO_*x*_ thickness leads to a reduction in the total current at the anode ($\text{Δ}i_{\text{disk}}<0$). This results from increased overpotential losses due to the insulating nature of the nanocoating and the reduced Cl^−^ concentration at the buried catalyst available to lead to CER, similarly to what was observed by Labrador et al. [29].

Conversely, the relative percentage change in OER molar selectivity shows an overall increase with increasing SiO_2_ nanocoating thickness for both the PE‐ALD (Figure 6D) and the SC (Figure 6F) coating methods. In contrast, with the T‐ALD method (Figure 6B), no OER molar selectivity improvement was measured at any tested thickness. The relative change in molar selectivity was shown as a reduction for the CER $\left(\text{Δ}S_{\mathrm{CER}}<0\right)$ and as an increase for the OER ($\text{Δ}S_{\mathrm{OER}}>0)$, following the same trends shown in Figure 1. In the case of the PE‐ALD deposition method (Figure 6D), an outlier V‐shaped region is present between 2 and 3 PE‐ALD cycles, where the poorest CER suppression occurs. This response is associated with the mechanical limitations of the fabricated anode itself, as discussed in the following paragraphs. At a comparable nominal SiO_*x*_ nanocoating thickness of 0.5 nm, as a value of comparison, PE‐ALD and SC methods resulted in a similar relative percentage decrease in CER molar selectivity ($\text{Δ}S_{\mathrm{CER}}\approx \text{−}36\%$) (Figure 6D,F). In contrast, the relative percentage increment in OER molar selectivity was slightly higher in the case of the PE‐ALD ($\text{Δ}S_{\mathrm{OER},\text{PE}\cdot \mathrm{ALD}}\approx \text{+}350\%$) compared to the SC ($\text{Δ}S_{\mathrm{OER},\mathrm{SC}}\approx \text{+}225\%$).

**FIGURE 7:**
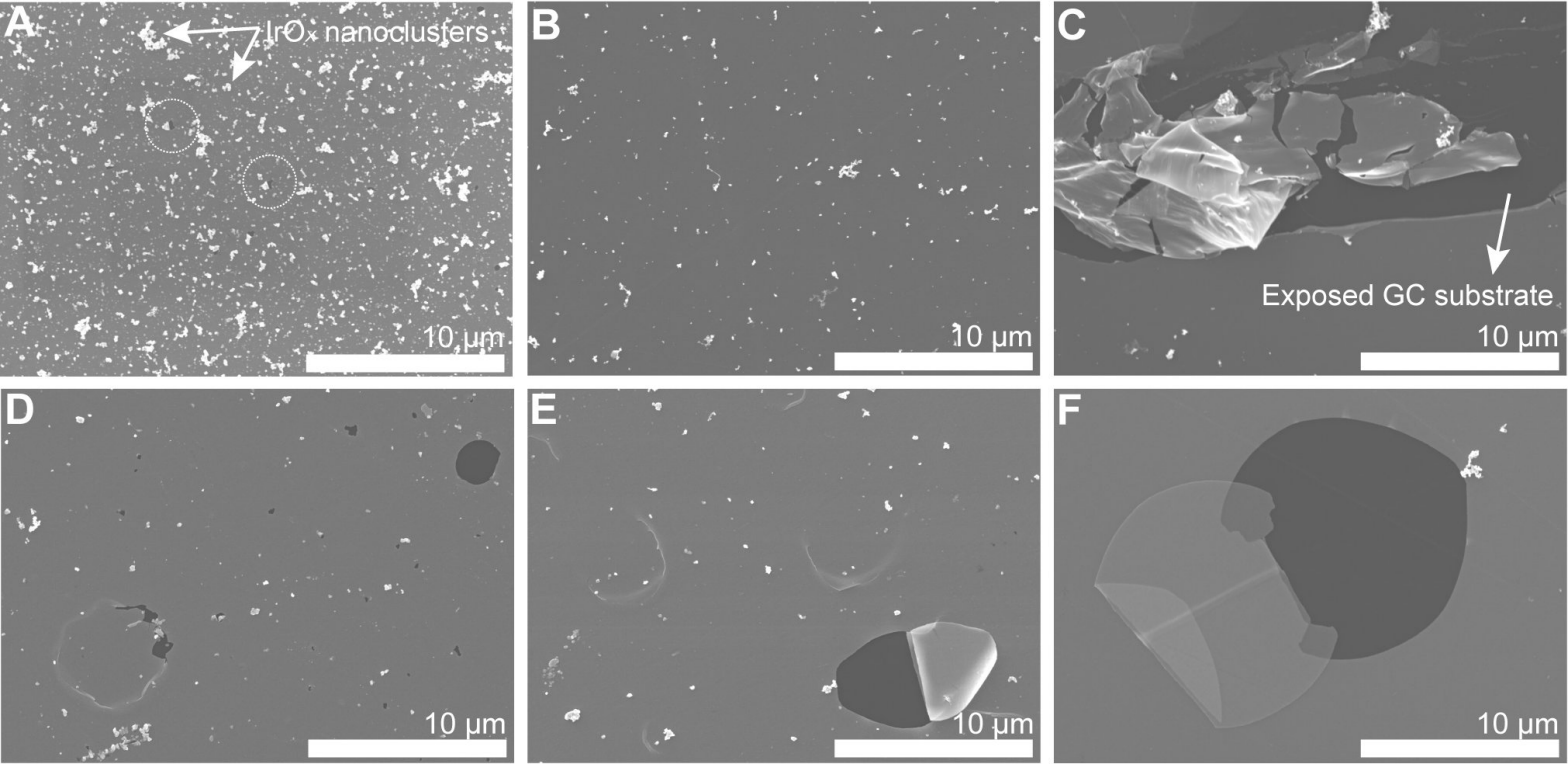
SEM images of postmortem anodes fabricated via PE‐ALD at 5*·*10^3^ magnification after 50 CV scans, corresponding to: (A) an uncoated anode (IrO_*x*_|GC) where small spots of catalyst delamination from the GC substrate are observed. The coated electrodes are displayed for an increasing number of ALD cycles as follows: (B) one cycle, (C) two cycles where co‐delamination of the catalyst coating system (SiO_*x*_|IrO_*x*_) from the GC substrate in the form of a black area is shown, (D) three cycles where an early stage of a blistering effect appears, (E) five cycles, (F) 10 cycles showing well‐defined blisters. In all electrodes, the white features correspond to Ir nanoclusters formed by the EDP of the catalyst. The SiO_*x*_ nanocoating is not visible in SEM images.

SEM images of postmortem electrodes reveal that thicker nanocoatings exhibit mechanical instability, leading to progressive co‐delamination of the SiO_*x*_|IrO_*x*_ stack from the GC substrate. This behavior is attributed to vigorous O_2_ and Cl_2_ evolution. Bubble nucleation and growth can amplify interfacial stresses and induce blistering, while bubble detachment and the resulting local electrolyte flow may accelerate catalyst layer delamination [65]. The uncoated anode (Figure 7A) exhibits some catalyst delamination, whereas the one‐cycle ALD sample (Figure 7B) shows no mechanical damage, indicating improved catalyst mechanical stability versus the uncoated control electrode. The first ALD cycles mark the beginning of nucleation island growth, as discussed in the LEIS analysis. The spaces between islands remain sufficiently separated to allow non‐resistive pathways for the release of gaseous products. As the number of cycles increases (Figure 7C,D), the islands start to coalesce into continuous patches. Gas accumulation beneath these regions may increase local stress and promote abrupt delamination. It is crucial to clarify that delamination in the PE‐ALD electrodes resulted in the detachment of both the coating and the underlying catalyst layer. This observation suggests that the adhesion at the SiO_*x*_|IrO_*x*_ interface exceeds that at the IrO_*x*_|GC interface, causing the coating and catalyst to detach together. Lastly, well‐defined blister zones are observed from five cycles onward (Figure 7E,F), likely due to a more uniform stress distribution that leads to a localized mechanical failure. The blistering effect was also observed for the SC and T‐ALD coating methods (Figures S15 and S16), supporting that the mechanical instability of the buried electrocatalyst nanocoating system increases with increasing coating thickness.

## 4 Conclusion

We show that the chloride‐blocking behavior of ultrathin SiO_*x*_ nanocoatings of comparable nominal thickness depends strongly on the deposition method. The tested SiO_*x*_ fabrication routes led to significantly different electrochemical outcomes. PE‐ALD and SC were associated with enhanced OER molar selectivity, whereas T‐ALD showed no apparent permselective effect under the investigated conditions. The Si 2p high‐resolution XPS peak expected at approximately 103.5 eV for stoichiometric SiO_2_ was shifted toward a lower binding energy for PE‐ALD and SC, consistent with silicon suboxides and possibly influenced by discontinuous coating coverage. This indicates that the tested deposition methods result in differences in the chemical environment and oxidation state of Si within the SiO_*x*_ coatings. Complementary apparent double‐layer capacitance and water contact angle measurements showed deposition‐dependent changes in interfacial charging. Apparent double‐layer capacitance and wettability increased for the individually evaluated PE‐ALD and SC electrodes, whereas an opposite trend was shown for T‐ALD electrodes. We further hypothesize that surface silanol groups, expected to be present based on the established surface chemistry of amorphous SiO_*x*_, may become protonated under acidic conditions and contribute to reduced Cl^−^ transport and enhanced OER selectivity. LEIS indicated that, under our conditions, ALD nanocoatings are better described as island‐like (non‐closed) films. We show that a discontinuous coating with an equivalent thickness of ~0.5 nm reduces the CER molar selectivity by up to ~36% compared with uncoated IrO_*x*_. Conversely, the relative percentage increase in OER molar selectivity was slightly larger in the case of the PE‐ALD ($\text{Δ}S_{\mathrm{OER},\mathrm{PE}\cdot \mathrm{ALD}}\approx \text{+}350\%$) compared to the SC ($\text{Δ}S_{\mathrm{OER},\mathrm{SC}}\approx \text{+}225\%$), relative to uncoated IrO_*x*_ electrode. The larger relative percentage reduction in CER current compared to OER current suggests a preferential hindrance of Cl^−^ transport, although the underlying mechanism remains to be fully elucidated. Additionally, increasing nanocoating thickness was observed to correlate with higher OER molar selectivity. Mechanical instability (blistering) of the electrocatalyst nanocoating assembly limited assessment of the intrinsic effect of coating thickness. Overall, the results indicate that CER suppression depends on coupled chemical, structural, and interfacial properties of the SiO_*x*_ coatings. Surface chemistry, coating coverage, wettability, transport through interisland gaps, and interfacial charging may all contribute to the electrochemical response. Therefore, the observed selectivity cannot be assigned to a single mechanism.

## Funding

This study was supported by Rijksdienst voor Ondernemend Nederland.

## Conflicts of Interest

The authors declare no conflicts of interest.

## Supporting information

Details of IrO*_x_* synthesis, electrophoretic deposition, SiO_2_ nanocoating deposition, and analytical techniques parameters are given. Additional X‐ray photoelectron spectrograms, apparent double‐layer capacitance measurements, wettability, and SEM images are provided in the Supporting Information. The authors have cited additional references within the Supporting Information [66, 67, 68].

## Data Availability

The data that support the findings of this study are available from the corresponding author upon reasonable request.
