# Supplementary material for: Effect of SiO x Nanocoating Deposition Route on Oxygen Evolution Reaction/Chlorine Evolution Reaction Selectivity of IrO x Anodes in Acidic Saline Water Electrolysis
Source: ChemSusChem. 2026 Sep 20;19(18):e71062. doi: 10.1002/cssc.71062 (PMC13591069; doi:10.1002/cssc.71062)
Supplement: Supplementary file 1 [file CSSC-19-e71062-s001.pdf]

Supporting Information

Effect of SiO_x Nanocoating Deposition Route on OER/CER Selectivity of IrO_x Anodes in Acidic Chloride Media

Katherine Stephanie Encalada-Flores[a], Koen de Graaf[a], Athina Tzavara-Roussi[b], Bhavesh Chavan[b], Philipp Brüner[c],Thomas Grehl[c], and J. Ruud van Ommen[b], Ruud Kortlever*[a]

[a] Process and Energy Department, Faculty of Mechanical Engineering
Delft University of Technology
Leeghwaterstraat 39, 2628 CB Delft, The Netherlands
E-mail: R.Kortlever@tudelft.nl

[b] Department of Chemical Engineering
Delft University of Technology
Van der Maasweg 9, 2629 HZ Delft, The Netherlands

[c] IONTOF GmbH
Heisenbergstraße 15, 48149 Münster, Germany

Table of Contents

S1. Experimental Procedures.....3

S1.1 Electrochemical set up components.....3

S1.2. Anode preparation before coating.....3

S1.3. SiO_2 nanocoating deposition via different fabrication methods.....4

Table S1. SiO_2 ALD Deposition Parameters.....5

S1.4. Quantification of Chlorine Evolution during Simultaneous OER and CER via RRDE.....5

Figure S1. CVs of Electropolished Pt Ring and Disk.....6

Figure S2. Selection of the Pt Ring Potential for RRDE Measurements.....6

Figure S3. RRDE Collection-Factor Determination.....7

Table S2. RRDE Collection-Factor Calculation.....7

Figure S4. Effect of Gas-Pocket Accumulation on RRDE Currents.....8

S1.5. Analytical techniques for the anode characterization.....9

S2. Additional characterization and Supplementary figures.....11

Figure S5. CVs of IrO_x before and after SiO_x Nanocoating.....11

Figure S6. Nominal SiO_x Nanocoating Thickness Calibration.....12

Figure S7. XRR Profiles of T-ALD-Coated IrO_x Electrodes.....13

Figure S8. FIB-SEM Cross-Section of a T-ALD-Coated IrO_x Electrode.....13

Figure S9. Si 2p XPS Spectra of SiO_2 Nanocoatings.....14

Figure S10. XPS Analysis of Residual Chlorine in T-ALD SiO_2.....14

Figure S11. Si 2p XPS Spectra before and after CER/OER Cycling.....15

Table S3. Double-Layer Capacitance before and after SiO_x Coating.....16

Figure S12. Coating-Induced Changes in Apparent Double-Layer Capacitance.....16

Figure S13. Apparent Double-Layer Capacitance Determination.....17

Figure S14. Contact Angles of Uncoated and SiO_x-Coated IrO_x Electrodes.....18

Figure S15. Postmortem SEM of T-ALD-Coated IrO_x Anodes.....19

Figure S16. Postmortem SEM of spray-coated IrO_x Anodes.....19

S1. Experimental Procedures

S1.1. Electrochemical set-up components:

VSP-300 Potentiostat from BioLogic coupled with the modulated speed rotator (MSR) from Pine Research. PEEK shrouds (exchangeable disk) for both RDE (E5TQPK Series) and RRDE (E6 Series) from Pine Research to connect to the rotating shaft: Low-volume RDE/RRDE glass cell from Pine Research.

Two different reference electrodes (RE) were used: a leak-free Ag/AgCl electrode purchased from Innovative Instruments Ltd, and a reverse hydrogen electrode (RHE), HydroFlex, purchased from Gaskatel. Platinum foil (thickness 0.1 mm and purity 99.99%) was purchased from Thermo Fischer as a counter electrode (CE). The working electrode consisted of SIGRADUR®G glassy carbon disks as substrate (5 mm diameter and 4 mm height) from HTW Hochtemperatur Werkstoffe GmbH, which were covered with IrO_X as catalyst and further protected with a SiO_X nanocoating.

S1.2. Anode preparation before coating

Glassy carbon (GC) substrate surface preparation

The brand-new GC disks were manually hand-polished using a 0.05 µm alumina slurry on a micro-cloth. For reused GC disks, a more aggressive polishing protocol was used, consisting of a first polishing with 5.0 µm alumina slurry on a nylon pad, followed by 0.3 µm and 0.05 µm alumina slurry, both on a micro-cloth pad. Later, the disks were rinsed with milli-Q water followed by sonication in isopropanol for 3 minutes to remove any traces from the polishing step.

$IrO_x \cdot nH_2O$ nanoparticles colloid solution preparation

This protocol was adapted from Zhao et al.[66] and Vos et al.[30] Sodium hydroxide (NaOH, 97%), sodium hexachloroiridate (IV) hexahydrate ($Na_2IrCl_6 \cdot 6\ H_2O$, 99.9% trace metals basis), perchloric acid ($HClO_4$, 70%), and sulfuric acid (H_2SO_4, 97%) were purchased from Honeywell. A 25 mL volume of 2 µM Ir solution was prepared by dissolving 28 mg of $Na_2IrCl_6 \cdot 6H_2O$ in 25 mL of 0.1 M NaOH solution. In this phase, the solution had a light yellow tinge and a pH of around 13.5. The solution was placed in a round-bottom flask coupled with a thermometer and a magnetic stirrer. It was immersed in a water bath and heated to 90 °C, then kept at that temperature for 20 minutes. Immediately thereafter, the round-bottom flask was placed in an ice bath until the solution reached approximately 0 °C, while stirring continuously. Once the ice temperature was reached, concentrated perchloric acid (70%) was added while stirring continuously to obtain a pH = 1. Once pH ≈ 1 was reached, the solution was stirred in the ice bath for one hour, allowing the condensation of hydrated IrO_x colloids. After one hour, 10 M NaOH was added while stirring continuously to obtain the desired pH≈ 1.5. A dark blue IrO_x colloid solution was ultimately obtained. The solution was either used immediately or kept in the fridge at 4 °C (pH≈ 1.5).

Catalyst electrophoretic deposition (EPD) of IrO_x nanoparticles onto GC disk using RDE

To form the IrO_x catalyst layer, previously synthesized IrO_x nanoparticles (in solution) were electrophoretic deposited onto a polished glassy carbon (GC) disk based on the protocol proposed in the supporting document by Vos et al.[30] The main modification in our protocol is

limiting the deposition process (during the chronoamperometry step) by the cut-off charge to 0.4 C as opposed to time. This ensures consistency in the amount of charge passed to the substrate, independent of small variations in concentration in the nanoparticle-containing solution. A three-electrode cell configuration was used where the GC disk, placed into the E5 Series RDE, performs as the WE, a leak-free Ag/AgCl as RE, and a Pt coil as CE. The solution was sonicated for at least 5 minutes to ensure no particle agglomeration and to bring the temperature to ambient, in case it had been previously stored in the fridge. The rotation of the RDE was set to 600 rpm for deposition. The protocol of deposition consists of ① 10 CV scans with a scan rate of 250 $mV \cdot s^{-1}$ between 0.16-1.36 V vs. Ag/AgCl, ② CA at 1.255 V vs. Ag/AgCl until $Q-Q_o=0.4$ C. This last step varied between 17 and 20 minutes, depending on each batch of solution. Contrary to what was reported by Vos et al.[30] a yellowish film was observed after the deposition.

Catalyst preconditioning and characterization before SiO_2 coating deposition using RDE

After electrophoretic deposition, the IrO_x catalyst layer was preconditioned to ensure that the catalyst is stabilized and achieves optimal electrochemical activity for OER. This was done in the RDE low-volume cell set-up filled with 12 mL electrolyte (0.5 M H_2SO_4), previously degasified by bubbling Ar gas for 5 min. Preconditioning was performed at a rotation rate of 1500 RPM. The protocol of deposition consists of ① 40 CV scans with a scan rate of 250 $mV \cdot s^{-1}$ in a potential range of 1.3 to 1.55 V vs. RHE. ② 3 CV scans with a scan rate of 50 $mV \cdot s^{-1}$ in a potential range of 0.0 to 1.4 V vs. RHE. The latest step enables a closer examination of the redox peaks of iridium and its stability during multiple cycles.

S1.3. SiO_2 nanocoating deposition via different fabrication methods

Thermal Atomic Layer Deposition (T-ALD)

T-ALD experiments were conducted in an in-house-built system featuring a tubular horizontal reactor chamber designed for flat substrates, with an integrated substrate holder for the GC substrates. The system works under atmospheric conditions. Silicon tetrachloride ($SiCl_4$, Alfa Aesar) served as a precursor, and a mixture of water vapor/ozone was used as a co-reactant. The co-reactant stream consists of water vapor from the bubbler and ozone generated by an aquarium-type ozone generator (Sander Certizon) supplied with synthetic air. The liquid chemicals, $SiCl_4$ and water, were delivered from the bubblers by a vapour-drawn technique. Ozone was used in the co-reactant mixture to promote complete oxidation and remove residual −Cl. N_2 (99.99% purity) was used both as carrier and purging gas.

Plasma Enhanced Atomic Layer Deposition (PE-ALD)

PE-ALD experiments were carried out in a commercial Fiji G2 instrument from Veeco. The system operates under high-vacuum conditions. Bis(diethylamino)silane (BDEAS, $C_8H_{22}N_2Si$, 97%, STREM Chemicals) served as the silicon precursor, and O_2 plasma was used as the oxidizing co-reactant. The precursor was introduced into the chamber via vapor-phase delivery. A remote plasma source operating at 300 W was used, with a gas mixture of oxygen (O_2) and argon (Ar). Ar served as the plasma excitation gas, while oxygen provided reactive species (e.g., O radicals) for surface activation and oxidation of the precursor. Ar was used both as a carrier and purging gas. Further details on the operating conditions for both of the ALD techniques can be found in **Table S1**.

Table S1. SiO_2 ALD Deposition Parameters. Deposition parameters for SiO_2 deposition by thermal atomic layer deposition (T-ALD) and plasma-enhanced atomic layer deposition (PE-ALD).

Conditions	T-ALD	PE-ALD
Reactor temperature [°C]	150[67,68]	100
Reactor pressure [mbar]	1013 (atmospheric)	10^{-7}
Plasma power [W]	N/A	300
Carrier gas	N_2	Ar

	Steps	Substance	Pulse length	Substance	Pulse length
**1**	Precursor dosing	$SiCl_4$	15	BDEAS	0.06
**2**	Purging	N_2	60	Ar	20
**3**	Co-reactant dosing	H_2O/O_3	30	O_2/Ar plasma	20
**4**	Purging	N_2	60	Ar	10

Spray coating (SC)

For the preparation of spray-coated SiO_2 layers, a precursor solution consisting of polydimethylsiloxane (PDMS, MW 410, Thermoscientific) dissolved in toluene acted as the Si source. Different concentrations of PDMS in toluene ($C_6H_5CH_3$, 99.8 %, Sigma-Aldrich), containing 2, 5, 10, and 20 mg·ml^{-1} PDMS in toluene, were prepared to investigate the effect on the coating. The GC|IrO_x disks, mounted on an aluminum holder placed on a heating plate at 120 °C, were spray-coated with the solution above. Spray coating was performed in a single pass using the precursor solution with an airbrush (0.3 mm nozzle, spray-work HG Single Airbrush, Tamiya). The disks were left on the heating plate for at least 5 minutes to allow all the toluene to evaporate before the curing step. To obtain SiO_2, the PDMS coating was chemically oxidized in a UV-ozone chamber for 2 h (PSD Pro Series Digital UV Ozone System, OES-1000D Ozone Elimination System).

S1.4. Quantification of Chlorine Evolution during Simultaneous OER and CER via RRDE

Platinum Electropolishing

Before starting the experiment, the Pt ring was electropolished in degasified 0.5 M H_2SO_4 electrolyte by performing 40 CV scans in a potential range from 0 to 1.5 V vs. RHE with a scan rate of 250 mV·s^{-1} to ensure a consistent baseline of the Pt ring surface for the chlorine reduction reaction (CRR). The resulting CV of the Pt ring is shown in **Figure S1**.

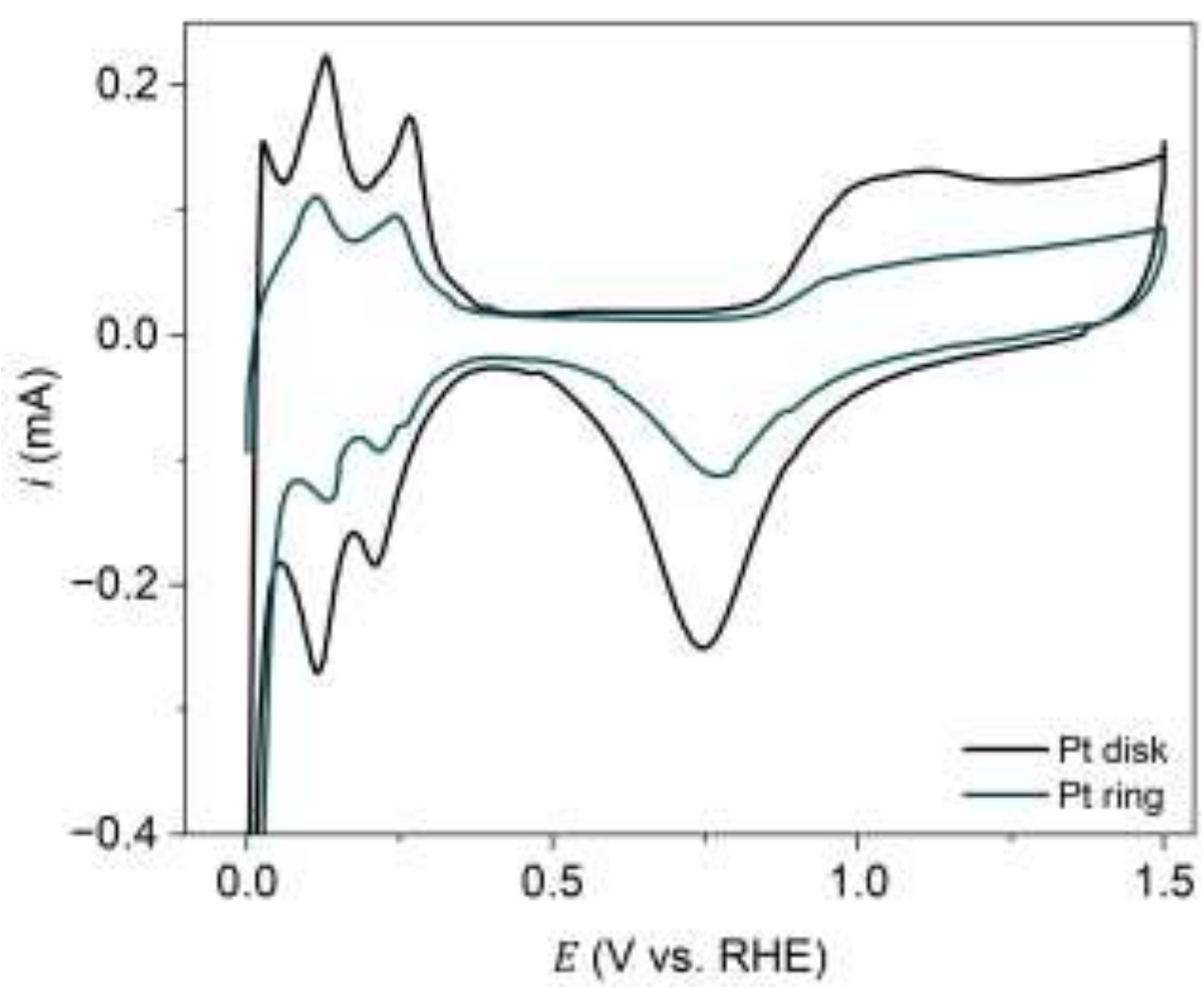

Figure S1*. *CVs of Electropolished Pt Ring and Disk. Representative CVs recorded after electropolishing of (i) the Pt ring prior to each RRDE quantification experiment and (ii) the Pt disk used for collection-factor determination.

Validation of Ring Potential Selection

At the selected ring potential, oxygen cannot be effectively reduced (ORR) as lower potentials are required. A controlled experiment was conducted applying potentials as low as 0 V vs. RHE, in a free-chloride containing electrolyte, showing no current signal at the ring (as shown in **Figure S1**). It is worth mentioning that only the forward scan of each CV scan was used for further analysis.

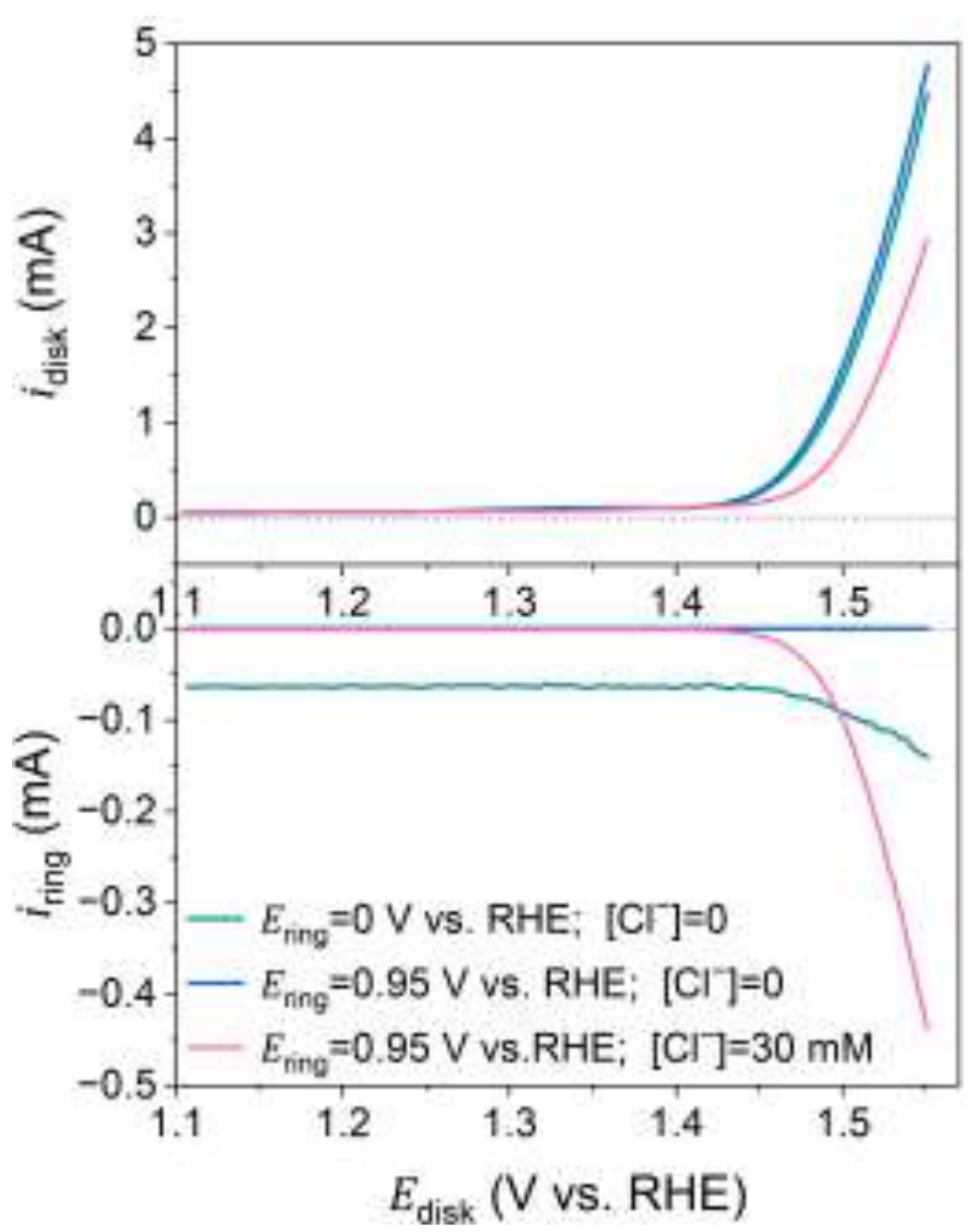

Figure S2. **Selection of the Pt Ring Potential for RRDE Measurements.** Disk and ring polarization curves corresponding to the 10th CV scan for an uncoated anode (IrO_x|GC) at different ring potentials. The ring current signal is zero at E_{ring}=0.95 V vs. RHE in a non-chloride containing solution. This demonstrates that no oxygen reduction reaction (ORR) occurs in the ring under the testing conditions of this study.

Experimental determination of the collection factor (*N)*

The determined collection factor of our RRDE system was *N*=0.237. As shown in **Table S1**, *N* remains constant within the ω range of interest, assuring stability of the products reaching the ring. Caution is advised when implementing strategies to avoid misreading the signal in the ring due to bubble shielding, as discussed in section 2.3. *N* was calculated using a Pt disk-Pt ring system in the same low-volume cell setup (0.5 M H_2SO_4 + 30 mM NaCl). Pt elements were previously electropolished to ensure a clean electrochemical surface. For the electrochemical protocol, the Pt disk was scanned once in a potential range from 0.8 to 1.67 V vs. RHE at different rotation speeds (ω) of 400, 900, and 1600 RPM while the ring potential was held constant at 0.95 V vs. RHE. Figure S3 and Table S2 show the current response of the system as a function of the disk potential.

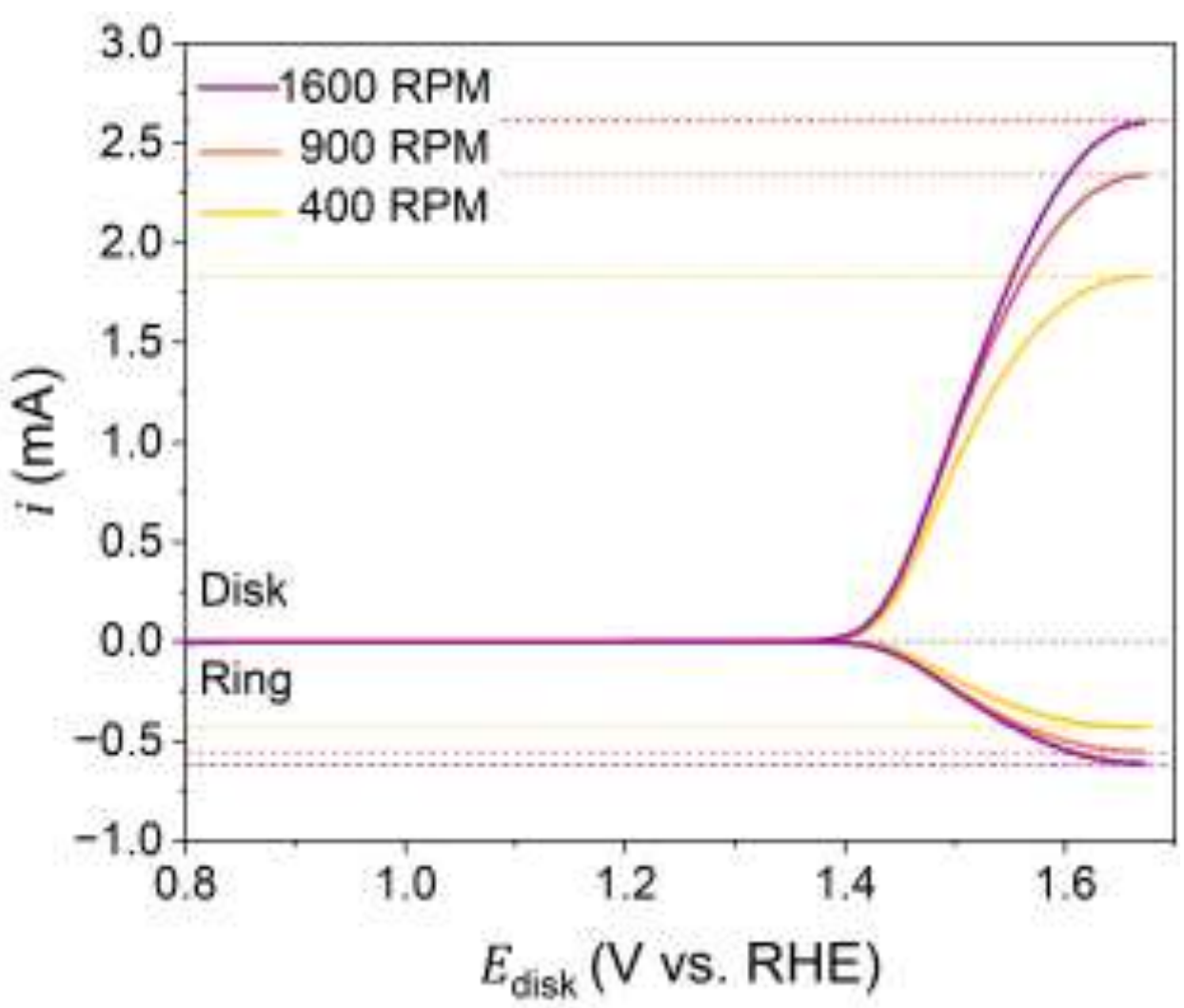

Figure S3. RRDE Collection-Factor Determination. Disk and ring current responses for determining the collection factor (N). The disk and the ring are platinum in an electrolyte of 0.5 M H2SO4 + 30 mM NaCl at different rotation speeds.

Table S2. RRDE Collection-Factor Calculation. Current values taken at 1.67 V vs. RHE extracted from Figure S3. to determine the collection factor (*N*) for the RRDE system.

**ω (rpm)**	i_{disk} **(mA)**	i_{ring} **(mA)**	***N*** $i_{\text{ring}}/i_{\text{disk}}$	***N* Average**	**STD.S**
**400**	1.83264	0.43389	0.236757	0.237074	0.00035
**900**	2.3424	0.55641	0.237457		
**1600**	2.61045	0.6187	0.237009		

Impact of Gas-Pocket Accumulation on Ring Current Response

Figure S4 shows the effect of gas-pocket accumulation within the Teflon spacer during RRDE measurements using an uncoated electrode. Between scans 1 and 25, the ring current progressively decreased and exhibited increased noise, while the disk current remained essentially unchanged. Since no catalyst coating was present, the observed decrease in ring current cannot be attributed to catalyst degradation. Instead, it is consistent with progressive obstruction of product transport to the ring electrode caused by gas accumulation in the spacer region.

Following the bubble-removal procedure, the ring current recovered abruptly (scan 26) and reached values comparable to or higher than those measured at the beginning of the experiment. This immediate recovery demonstrates that the apparent signal loss originated from gas-pocket accumulation rather than from irreversible changes in the electrode response. These results confirm that bubble removal restores the original measurement conditions and does not introduce an artificial change in the measured selectivity.

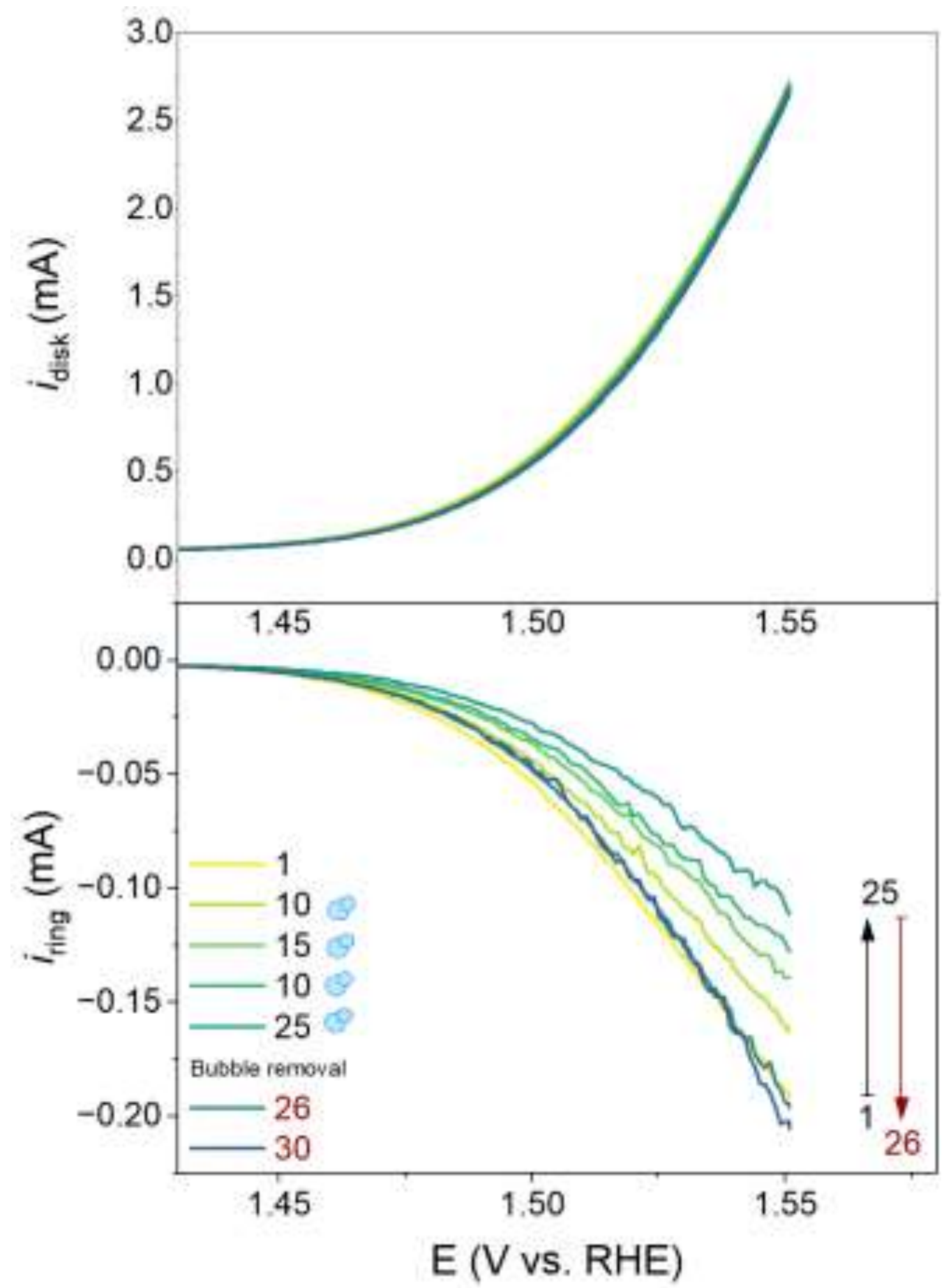

Figure S4. Effect of Gas-Pocket Accumulation on RRDE Currents. Evolution of disk and ring currents for an uncoated RRDE during gas-pocket accumulation in the Teflon spacer. Progressive suppression and increased noise of the ring current are observed between scans 1 and 25, while the disk current remains essentially unchanged. Bubble removal after scan 25 restores the original ring-current response (scan 26).

S1.5. Analytical techniques for the anode characterization

XPS

X-ray photoelectron spectroscopy (XPS) measurements were performed using a Thermo Fisher Scientific instrument equipped with a monochromatic Al Kα X-ray source and a flood gun for charge compensation. Survey spectra were first acquired to identify all elements present on the sample surface. High-resolution spectra were subsequently collected for the C 1s, Si 2p, Cl 2p, Ir 4f, and O 1s regions at a minimum of two different locations per sample. For XPS mapping measurements, a circular area 4.5 mm in diameter was analyzed. At least 49 acquisition points were distributed in a radial pattern across the selected area to evaluate the spatial distribution of the surface elements. The XPS spectra were processed using Thermo Avantage software. Elemental and peak assignments were made using the XPS Knowledge Library available in the Thermo Avantage database[52]. All spectra were calibrated by referencing the adventitious carbon C 1s peak to a binding energy of 284.8 eV.

LEIS

The coverage of SiO_2 ALD-fabricated nanocoatings was determined via the Qtac 100 high Performance Low Energy Ion Scattering by Iontof equipped with a double toroidal energy analyzer. All samples analyzed for this report were pre-cleaned in vacuum by exposure to atomic oxygen from a remote plasma source to remove volatile adsorbates, which would otherwise cover the surface of interest. Two types of ion beams were used, one using ${}_{4}He^{+}$as the ion species and the other ${}_{20}Ne^{+}$. Using Ne^{+} as the analysis ion, only the heavy element Ir can be measured, but with better sensitivity compared to He^{+} measurements. All spectra shown are sum spectra of two different analysis spots per sample, 2 x 2 mm^2 per spot.

SEM

Scanning electron microscopy (SEM) was conducted using a JEOL IT800SHL microscope integrated with a secondary electron (SE) detector. The acceleration voltage was 5kV, kept low to optimize surface detail, as we were targeting ultrathin coatings. The working distance was 10 mm.

Ellipsometric Spectrometry

We approximated the SiO_2 nanocoating thicknesses for all fabrication methods by placing a witness Si wafer alongside the GC anodes during nanocoating deposition for all tested fabrication methods. A J. A. Woollam alpha-SE ellipsometer. The thickness values were obtained by fitting a two-layer optical model in which the native SiO_2 layer of the uncoated Si wafer was subtracted. The wafers were covered with a thin native oxide (SiO_2) layer and did not undergo any specific cleaning steps.

Attempts to directly determine the coating thickness on the catalyst electrodes using atomic force microscopy (AFM), focused ion beam-scanning electron microscopy cross-sections (FIB-SEM), and X-ray reflectometry (XRR) were inconclusive. AFM measurements were dominated by the intrinsic nanoscale roughness of the IrO_x surface and imaging artifacts, FIB-SEM cross-sections did not allow reliable visualization of the coating due to structural damage of the porous

catalyst layer during milling, and XRR analysis was hindered by the high surface roughness, preventing robust multilayer fitting and accurate thickness extraction. Further details are provided in Figures S7 and S8.

Apparent double-layer capacitance measurements

The apparent double-layer capacitance, $C_{\mathrm{dl,app}}$, was determined by cyclic voltammetry in Ar-purged 0.5 M H_2SO_4. A three-electrode cell configuration was used, in which the GC disk, placed in the E5 Series RDE, served as the WE, the RHE as the RE, and a Pt foil as the CE, no rotation was applied. Measurements were conducted within a potential window from 0.14 to 0.20 V vs. RHE, in which no visible major faradaic features were observed under the experimental conditions. Before the scan-rate-dependent measurements, each electrode was stabilized by performing at least 50 consecutive potential cycles at 100 mV·s^{-1}. Subsequently, 10 consecutive cycles were recorded at scan rates of 100, 50, 30, 20, 10, and 5 mV·s^{-1}, in descending order. The tenth cycle recorded at each scan rate was selected for the capacitance analysis. The anodic and cathodic currents were extracted at 0.17 V vs. RHE and plotted separately as a function of scan rate. Linear regressions were applied to the anodic and cathodic current responses, and $C_{\mathrm{dl,app}}$was calculated as the average of the absolute values of the corresponding slopes, as shown in equation (s.1):

$$C_{\mathrm{dl,app}} = \frac{|\, m_{\mathrm{anodic}} \,| + |\, m_{\mathrm{cathodic}} \,|}{2} \qquad \text{(s.1)}$$

where $m_{\mathrm{anodic}}$and $m_{\mathrm{cathodic}}$are the slopes obtained from the anodic and cathodic current versus scan-rate plots, respectively. Measurements were performed on the same electrode before and after deposition of the nominally 0.5 nm SiO_X coating. The relative percentage change in apparent double-layer capacitance can be calculated with equation (s.2):

$$\boldsymbol{\Delta C_{\mathrm{dl}}} = \left(\frac{C_{\mathrm{dl,app,coated}} - C_{\mathrm{dl,app,uncoated}}}{C_{\mathrm{dl,app,uncoated}}}\right) \cdot \mathbf{100}\ \% \qquad \text{(s.2)}$$

One electrode was measured per deposition method before and after coating; therefore, the reported uncertainties represent linear fitting errors rather than sample-to-sample variability.

Water contact-angle measurements

The apparent water contact angle was measured in air by the sessile-drop method using an OCA 25 contact-angle instrument (DataPhysics Instruments GmbH) equipped with SCA 20 U software (version 5.0.41, build 5041). A 1 µL of milli-Q water droplet was dispensed onto the electrode surface at a dosing rate of 1.00 µL s^{-1}, and the contact angle was determined immediately after droplet deposition at the three-phase contact line. Measurements were performed on uncoated IrO_x electrodes and electrodes modified with SiO_x coatings of approximately 0.5 nm nominal thickness prepared by T-ALD, PE-ALD, and SC. Two independently prepared samples were evaluated for each condition, and the results are reported as the mean standard deviation.

S2. Additional characterization and Supplementary figures

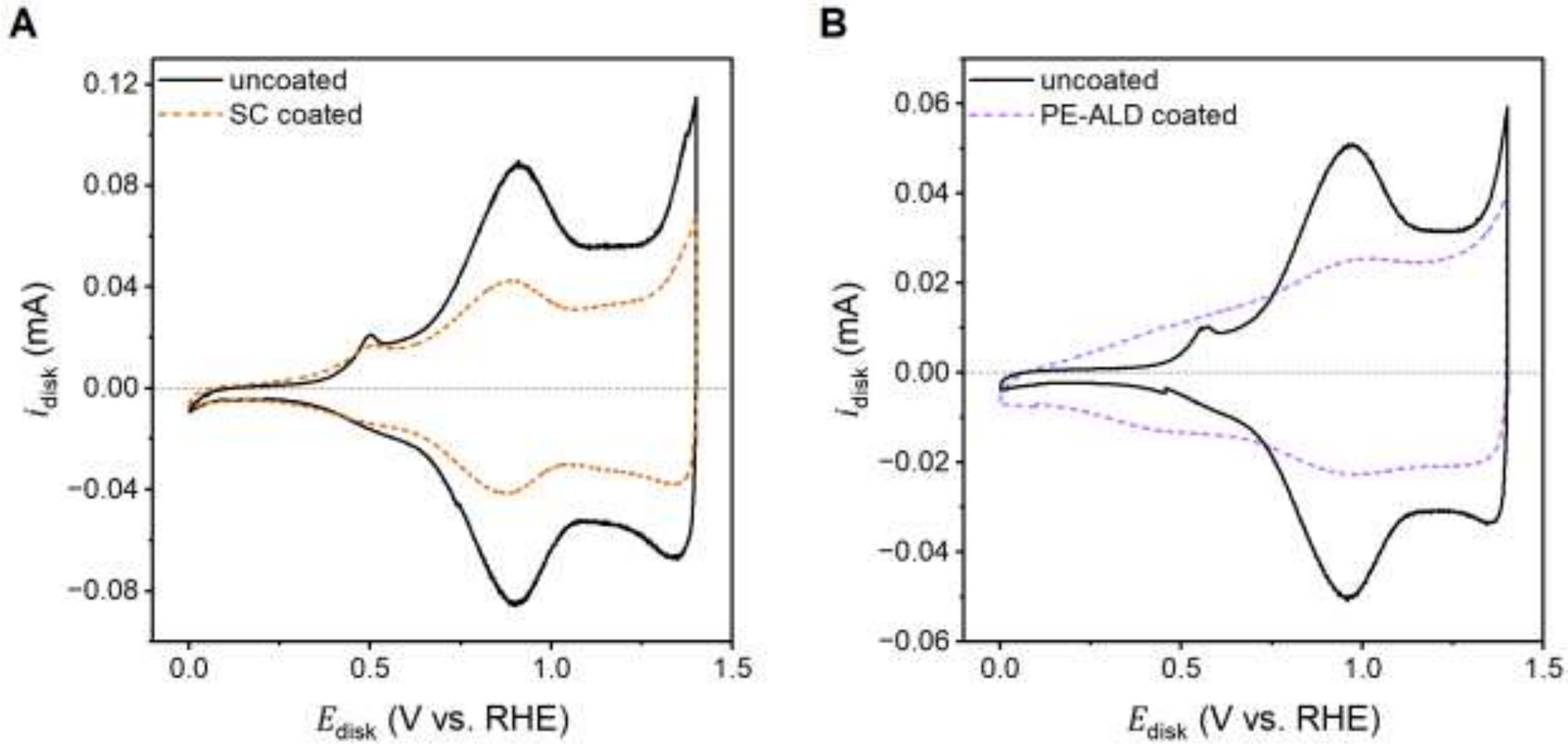

Figure S5. CVs of IrO_x before and after SiO_x Nanocoating. Cyclic voltammogram of IrO_x catalyst before and after SiO_x nanocoating for fabrication techniques of **(A)** SC corresponding to a sample coated with 20mg·mL^{-1} and **(B)** PE-ALD corresponding to 3 cycles.

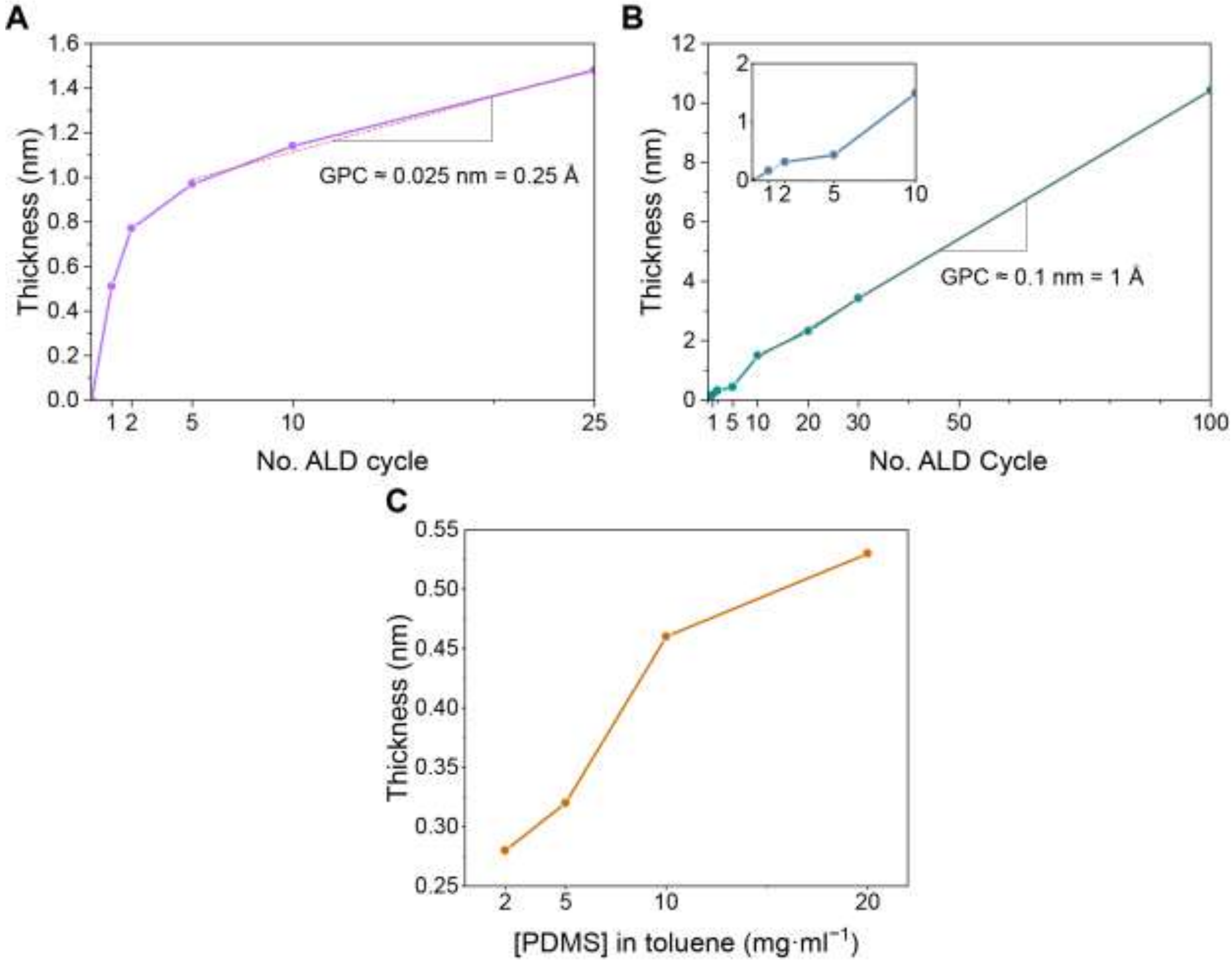

Figure S6. Nominal SiO_x Nanocoating Thickness Calibration. Thickness vs. increasing number of ALD cycles for **(A)** T-ALD and **(B)** PE-ALD and increasing precursor solution concentration for **(C)** SC fabrication method. A linear fit yields a growth per cycle (GPC) of 0.25 Å for T-ALD and 0.1 Å for PE-ALD. The thickness values correspond to a witness Si wafer coated along with the fabricated anodes. The reported thickness values were determined on simultaneously coated Si-wafers|SiO_2 witness wafers because their smooth surface allows accurate ellipsometric fitting. In contrast, direct ellipsometry on the IrO_x electrodes is not straightforward, as the rough and porous catalyst morphology, together with the lack of an established optical model and corresponding database parameters for the IrO_x layer, prevents reliable extraction of film thickness. This thickness as referred as “nominal” for the corresponding anodes.

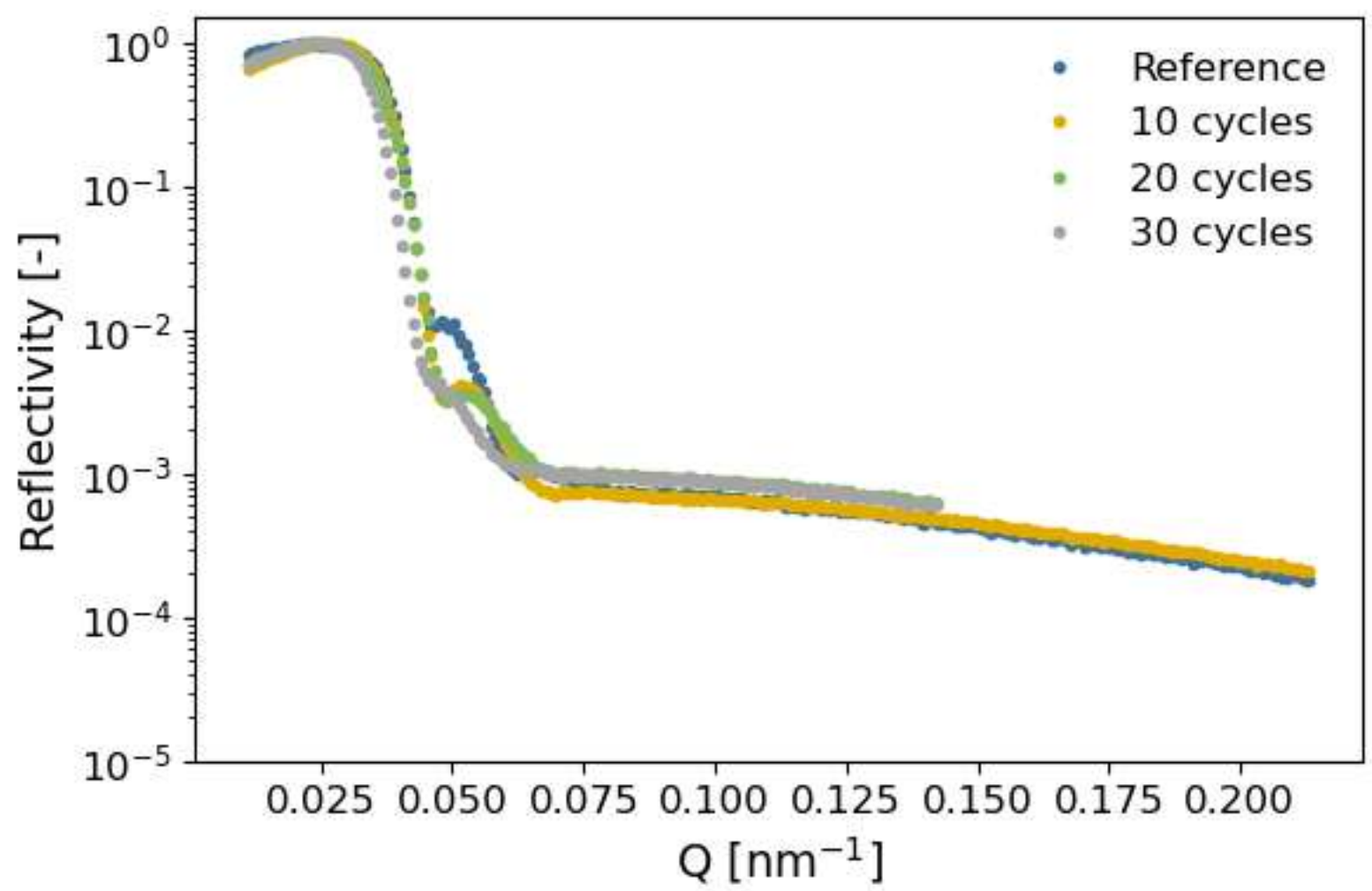

Figure S7. **XRR Profiles of T-ALD-Coated IrO_x Electrodes.** X-ray reflectometry (XRR) profiles of IrO_x electrodes coated by T-ALD using different numbers of ALD cycles. Differences in the critical-edge region near-edge region ($Q \approx 0.035–0.060\ \mathrm{nm}^{-1}$) are consistent with progressive surface modification with increasing cycle number. However, the rough and nanostructured IrO_x catalyst layer causes substantial diffuse scattering and suppresses distinct interference features. Consequently, reliable multilayer fitting and accurate determination of the IrO_x catalyst-layer and SiO_x coating thicknesses were not possible.

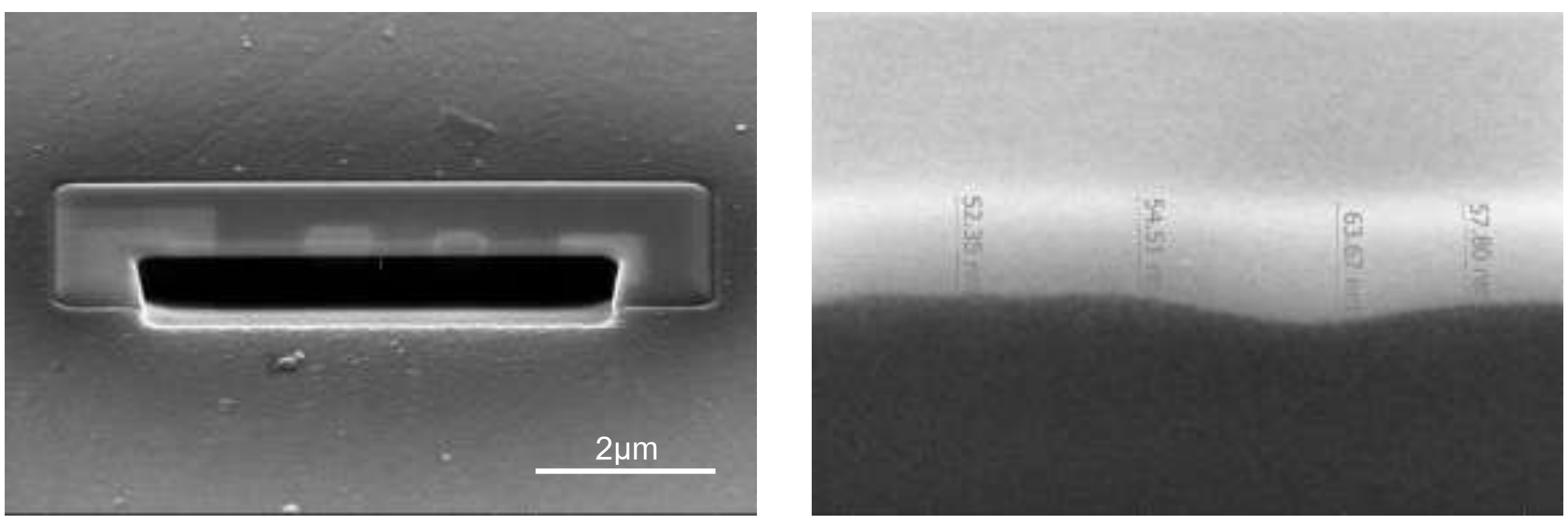

Figure S8. **FIB-SEM Cross-Section of a T-ALD-Coated IrO_x Electrode.** FIB-SEM cross-sectional analysis of an IrO_x electrode coated with SiO_x by 100 T-ALD cycles. **(A)** Overview of the focused ion beam trench prepared after deposition of a protective tungsten (W) layer. **(B)** Magnified cross-sectional image of the W|electrode interface. The high roughness and porosity of the catalyst layer prevented the clear identification of distinct layer boundaries. A contrast feature approximately 50-60 nm thick may correspond to the IrO_x catalyst layer; however, neither the IrO_x layer thickness nor the SiO_x coating thickness could be determined reliably from the cross-section, despite the use of a protective W layer before milling. These results illustrate the limitations of direct cross-sectional thickness measurements for this highly rough and porous electrode architecture.

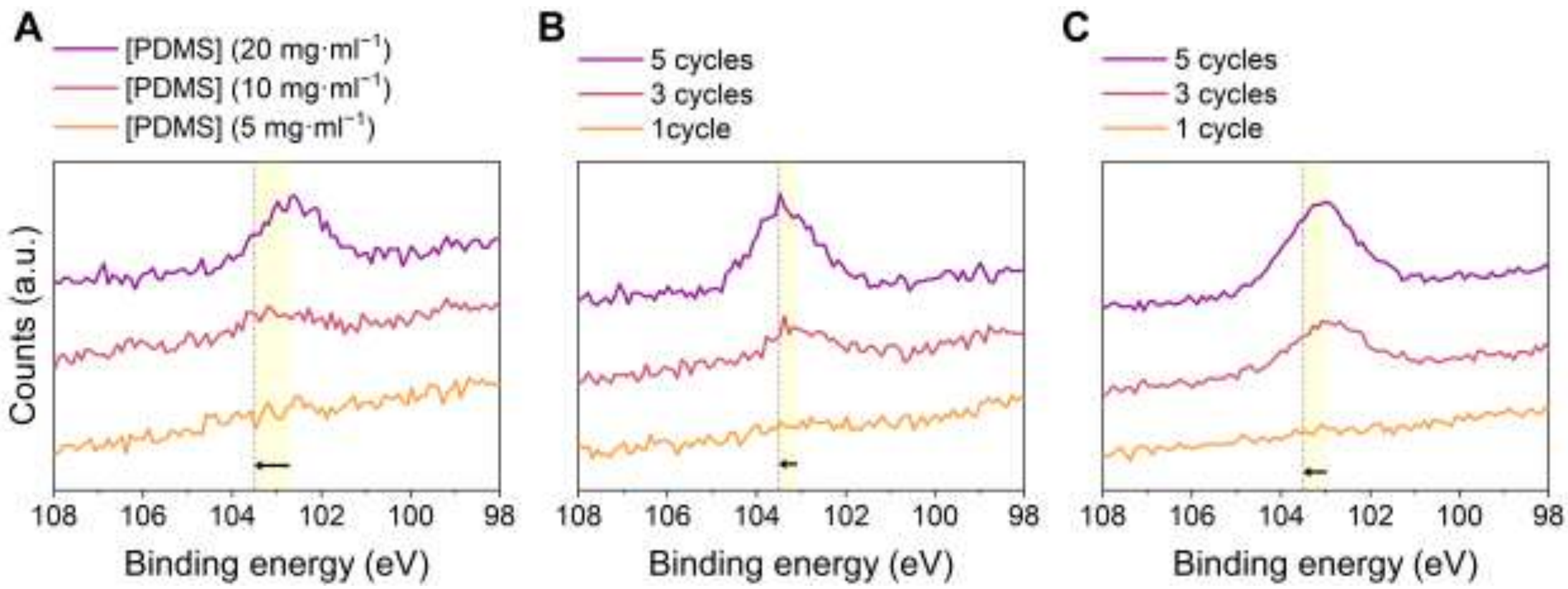

Figure S9. Si 2p XPS Spectra of SiO_2 Nanocoatings. XPS high-resolution spectra of the Si 2p peak for increasing SiO_2 thickness for various deposition techniques **(A)** SC, **(B)** T-ALD, and **(C)** PE-ALD. All spectra are referenced to the adventitious C 1s peak at 284.8 eV. A slight shift towards lower binding energies, relative to the expected 103.5–103.6 eV, is observed in all cases. As the layer becomes thicker, the shifting becomes less pronounced.

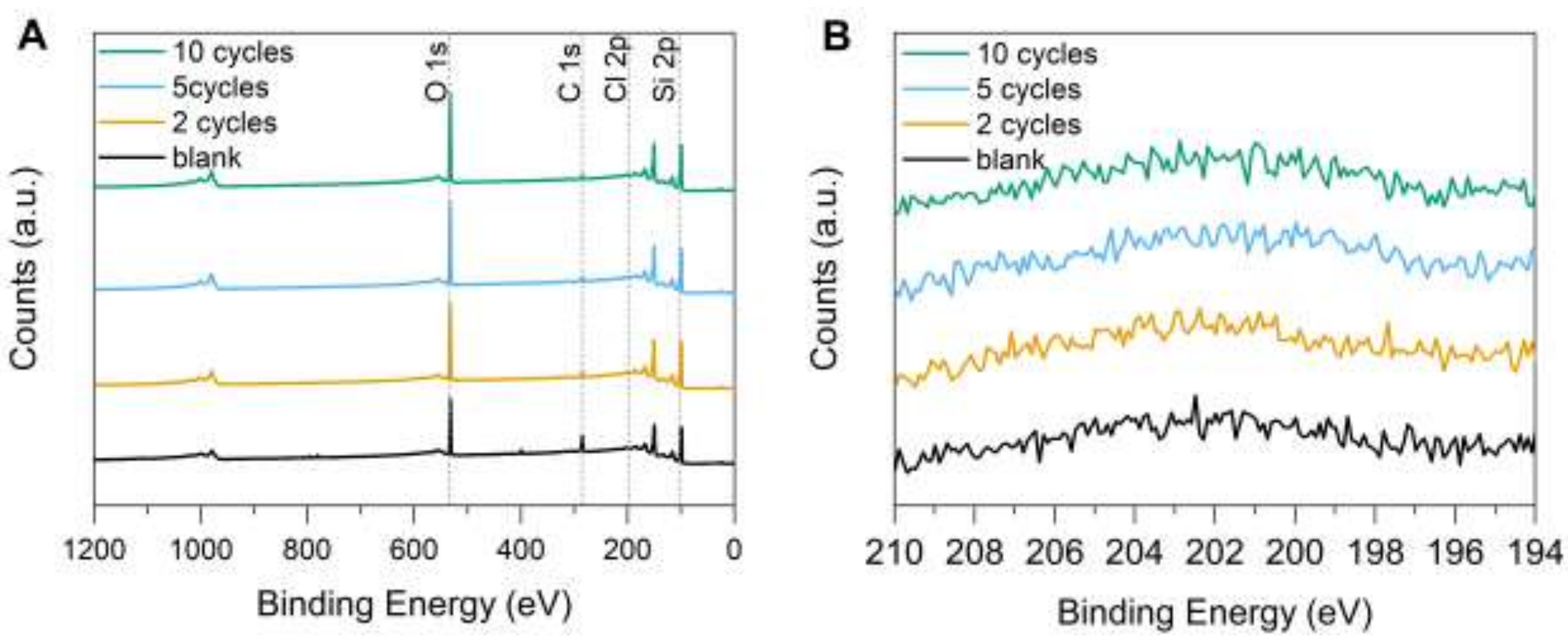

Figure S10. XPS Analysis of Residual Chlorine in T-ALD SiO_2. (A) XPS survey and **(B)** High-resolution spectra of the Cl 2p3/2 peak for increasing SiO_2 thickness for various deposition T-ALD technique on a Si wafer. High-resolution Cl 2p spectra show no doublet peak in the 198–200 eV range, indicating the absence of surface chlorine above the detection limit.

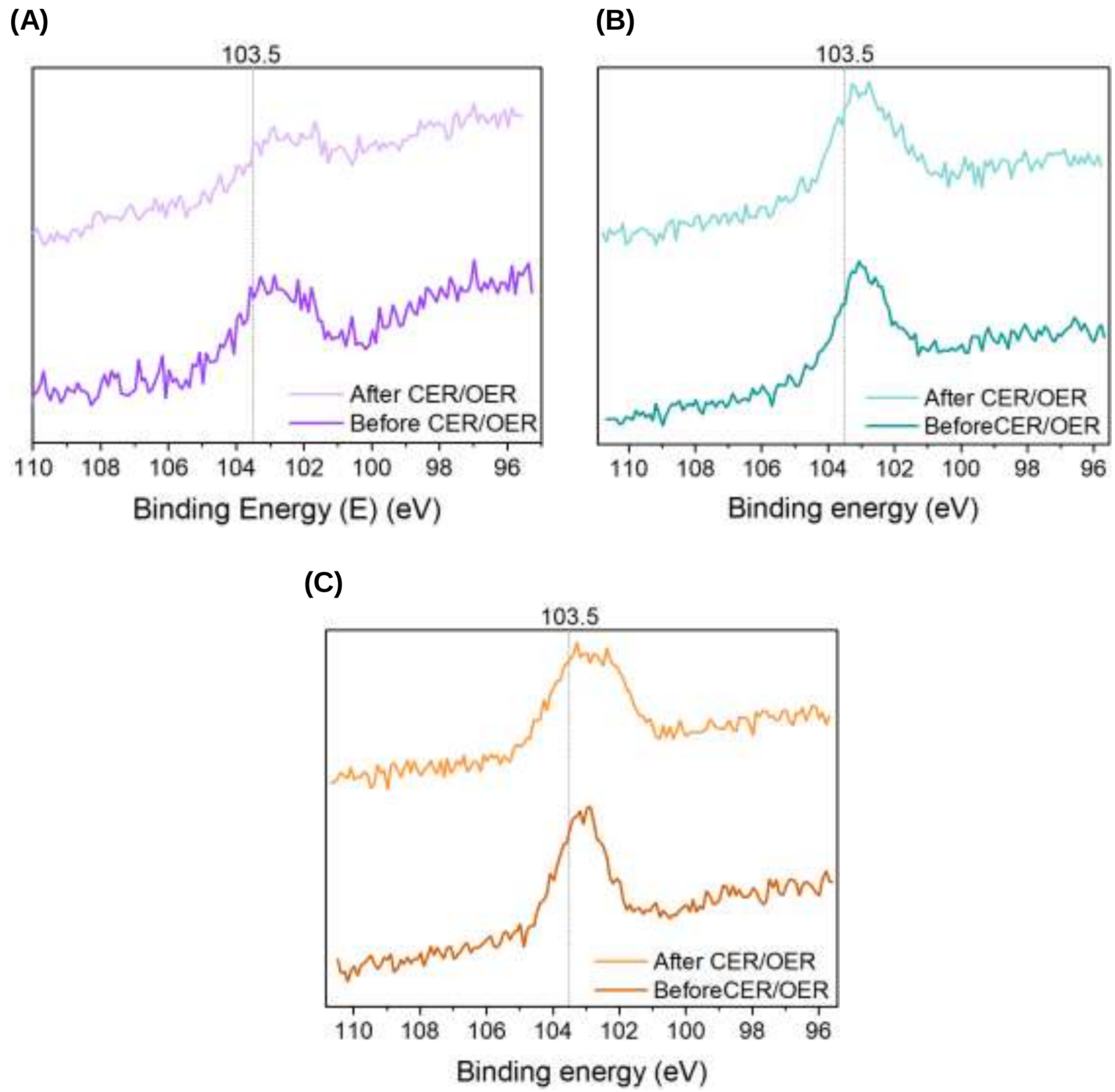

Figure S11. **Si 2p XPS Spectra before and after CER/OER Cycling.** XPS high-resolution spectra of the Si 2p peak for SiO2 coating before and after CER/OER reaction for various deposition techniques (A) T-ALD, (B) PE-ALD, and (C) SC. All spectra are referenced to the adventitious C 1s peak at 284.8 eV. A slight shift towards lower binding energies, relative to the expected 103.5 eV, is observed in all cases. The Si 2p signal remained near or below the binding energy expected for stoichiometric SiO_2, and no new Si-containing component was clearly resolved after cycling.

Table S3. Double-Layer Capacitance before and after SiO_x Coating. Apparent double-layer capacitance of IrO_X electrodes before and after deposition of nominally 0.5 nm SiO_X coatings by different deposition routes.

Deposition route	$C_{\mathrm{dl,app,uncoated}}$ (μF)	$C_{\mathrm{dl,app,coated}}$ (μF)	$\Delta C_{\mathrm{dl,app,coated}}$ %
**T-ALD**	25.55 ± 0.92	14.22 ± 0.85	-44.3
**SC**	19.73 ± 0.55	22.77 ± 0.46	+15.4
**PE-ALD**	22.42 ± 0.38	30.87 ± 0.98	+37.7

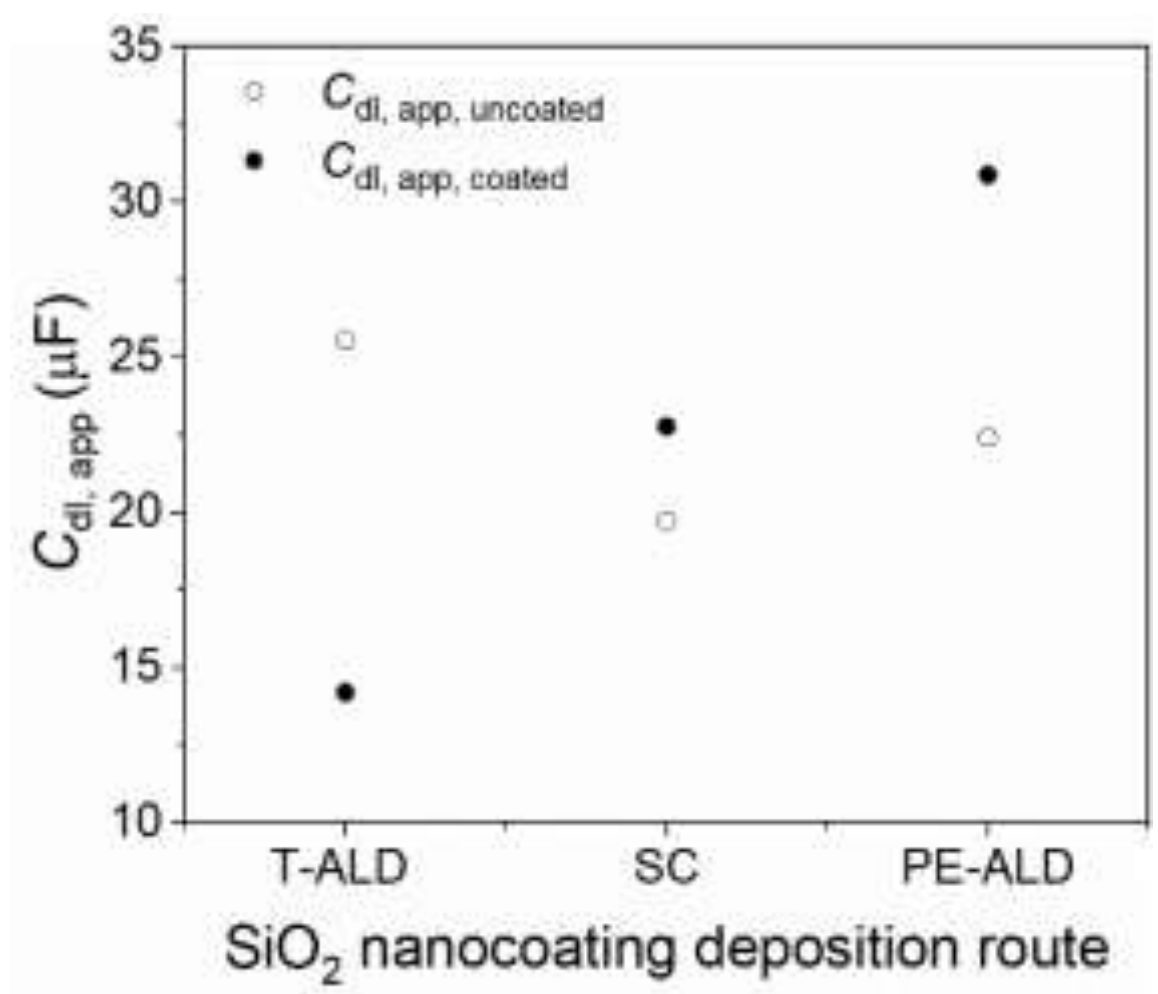

Figure S12. Coating-induced changes in apparent double-layer capacitance. Apparent double-layer capacitance, $C_{\mathrm{dl,app}}$, measured for individual IrO_x electrodes before and after deposition of nominally 0.5 nm SiO_x coatings by T-ALD, SC, and PE-ALD. Open and filled symbols represent the uncoated and coated states, respectively. The apparent double-layer capacitance was measured on the same electrode before and after coating. One electrode was evaluated for each deposition method. An example of a complete cyclic voltammograms and linear regressions are provided in Figure S13.

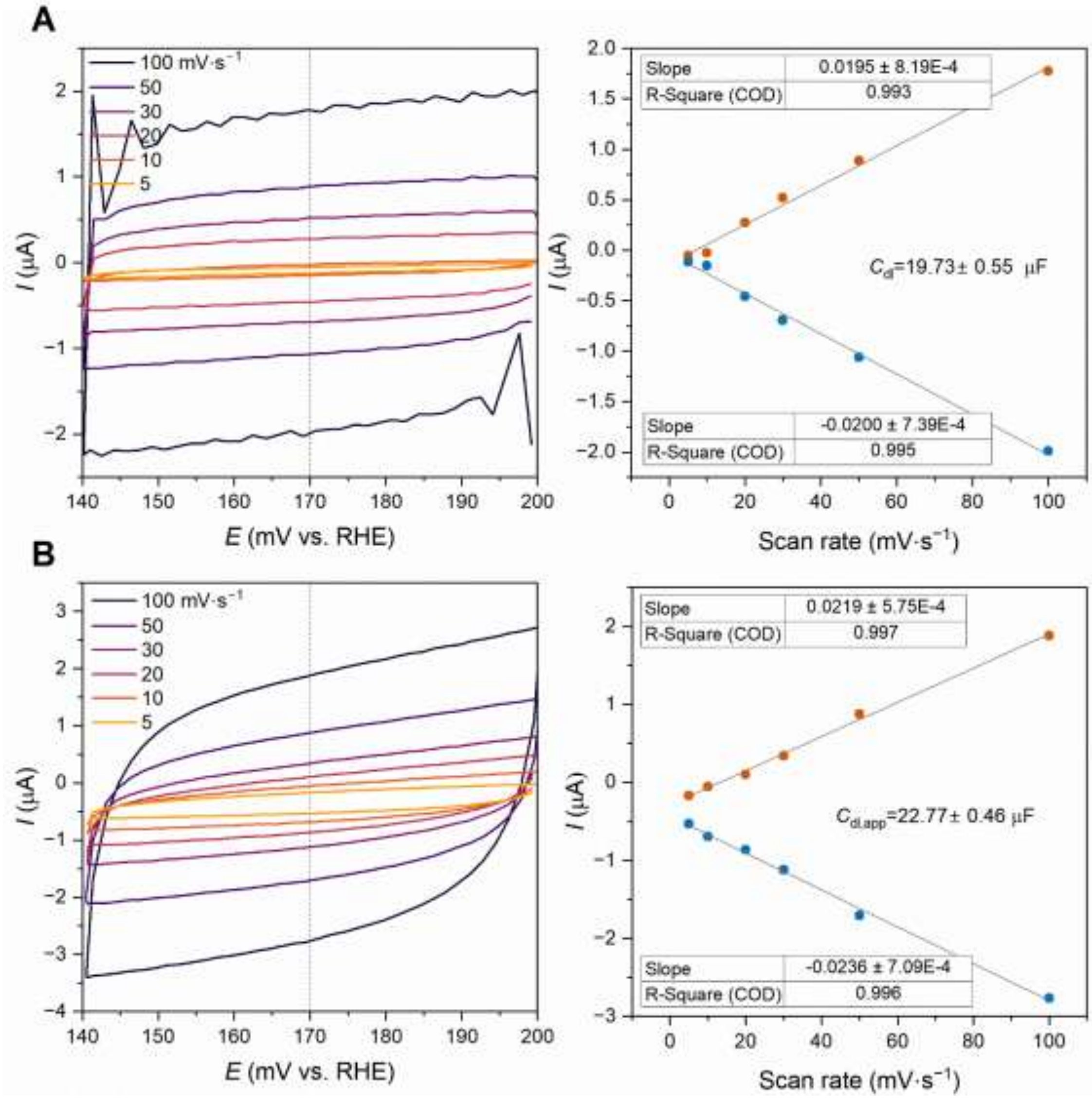

Figure S13. Apparent Double-Layer Capacitance Determination. Determination of the apparent double-layer capacitance for an **(A)** uncoated and **(B)** PE-ALD-SiO_2-coated IrO_x electrode. Cyclic voltammograms were recorded in Ar-purged 0.5 M H_2SO_4 over the potential window from 0.14 to 0.20 V vs. RHE, where no visible faradaic features were observed under the measurement conditions. Different scan rates of 100, 50, 30, 20, 10, and 5 mV·s^{-1}, and the tenth cycle at each scan rate was used for the capacitance analysis. The anodic and cathodic currents measured at 0.17 V vs. RHE were plotted separately as a function of scan rate. $C_{\mathrm{dl,app}}$was calculated as the average of the absolute values of the slopes obtained from the anodic and cathodic linear regressions. The results represent paired measurements performed on a single electrode before and after coating, with no independently prepared replicate electrodes. The reported uncertainties were derived from the linear regressions and therefore represent fitting uncertainty rather than sample-to-sample variability.

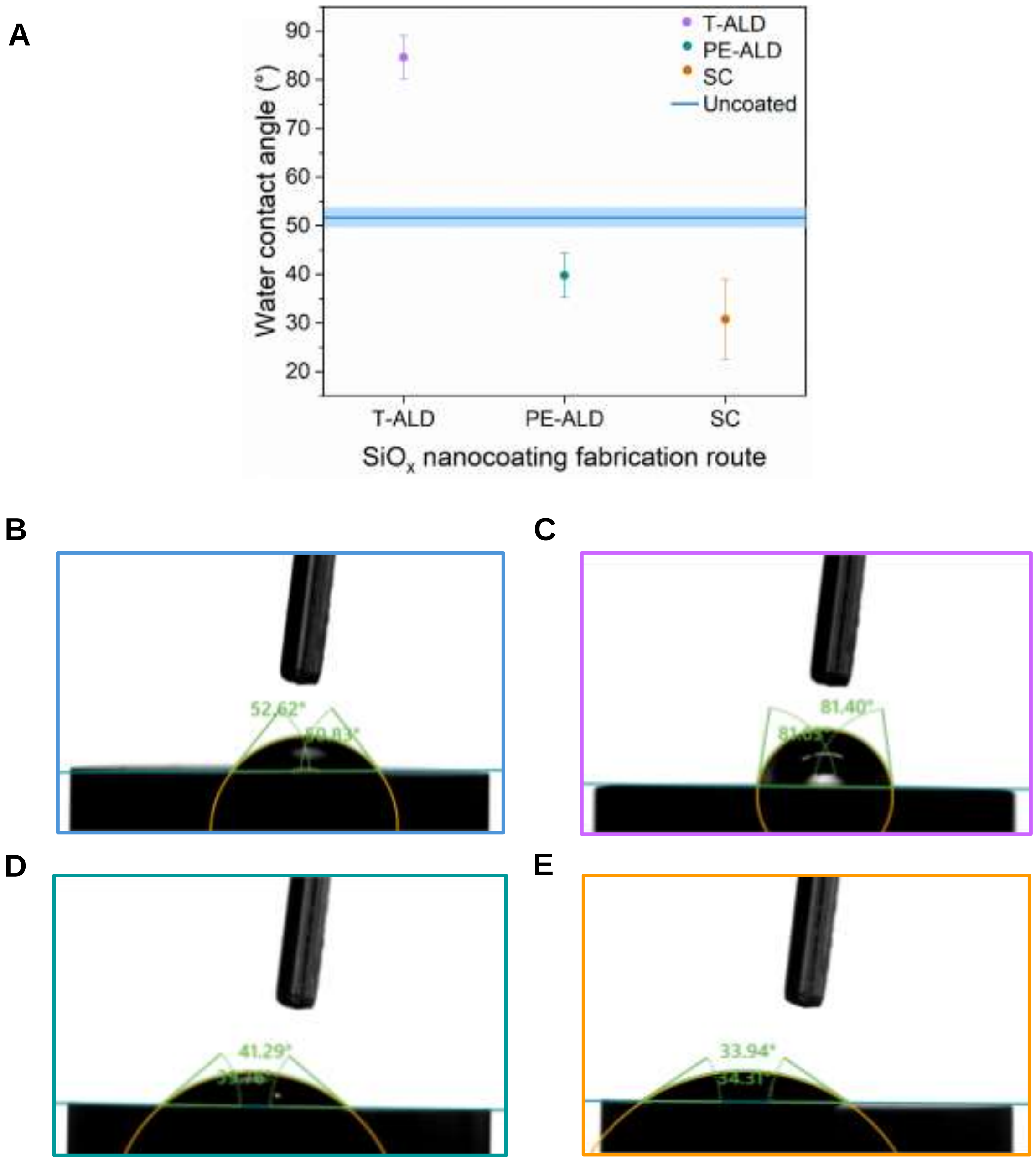

Figure S14. Contact Angles of Uncoated and SiO_x-Coated IrO_x Electrodes. (A) Contact angles measured for uncoated IrO_x electrodes and electrodes coated with SiO_x of approximately 0.5 nm nominal thickness by T-ALD, PE-ALD, and SC. Representative sessile-drop images are shown for **(B)** uncoated IrO_x, **(C)** T-ALD, **(D)** PE-ALD, and **(E)** SC electrodes. Symbols and error bars in panel A represent the mean and standard deviation, respectively, of two independently prepared samples. The blue line and shaded region represent the mean and standard deviation, respectively, of the uncoated IrO_x electrodes.

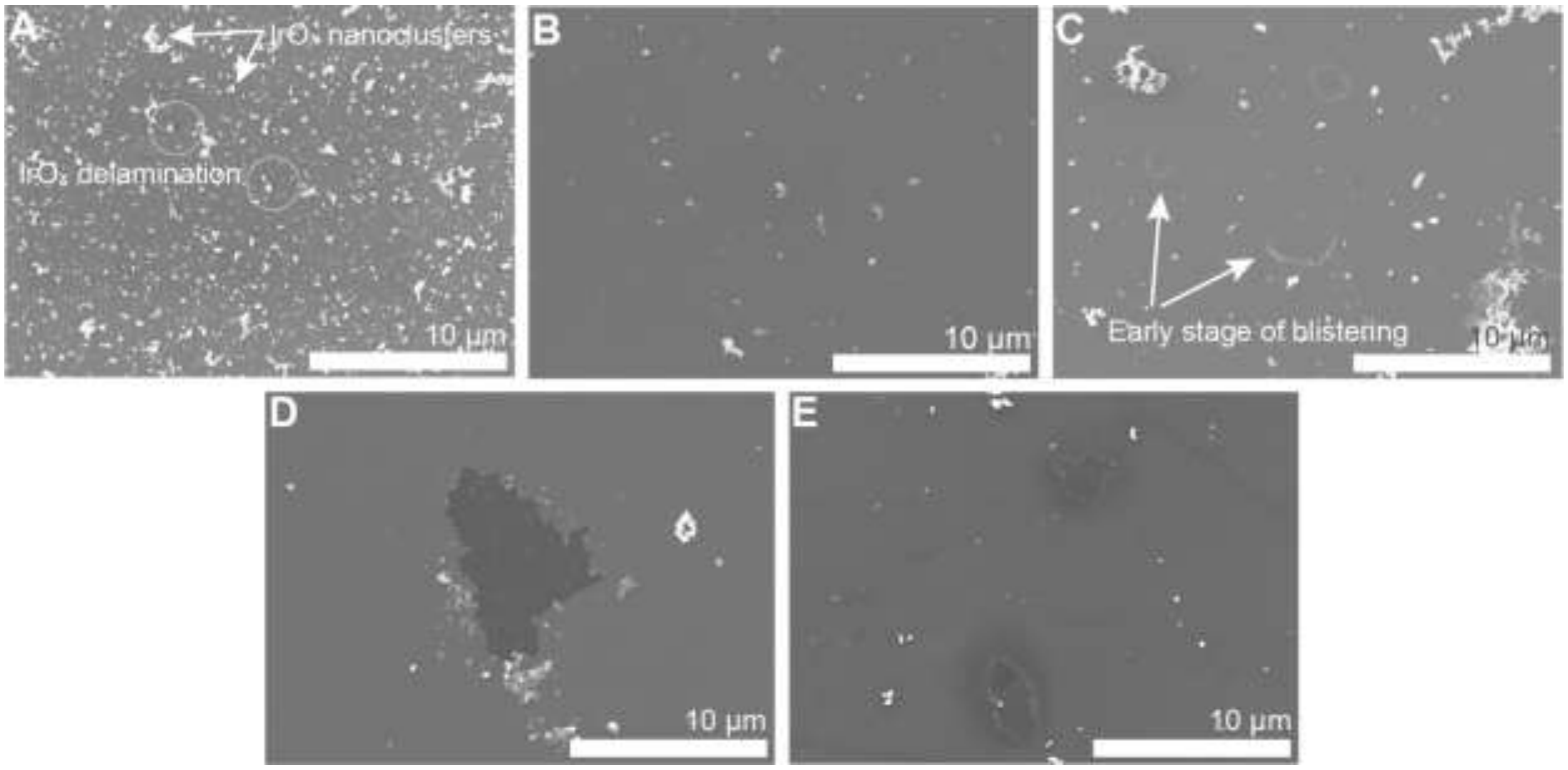

Figure S15. Postmortem SEM of T-ALD-Coated IrO_x Anodes. SEM images of postmortem anodes fabricated via **T-ALD** at $5{\cdot}10^3$ magnification after 50 CV scans, corresponding to **(A)** uncoated anode (IrO_x|GC) where small spots of catalyst delamination from the GC substrate are observed. The coated samples are displayed for an increasing number of ALD cycles as follows: **(B)** 1 cycle, **(C)** 2 cycles, where an early stage of blistering effect appears, **(D)** 3 cycles and **(E)** 5 cycles show co-delamination of the catalyst-coating system (SiO_x|IrO_x) from the GC substrate in the form of black spots.

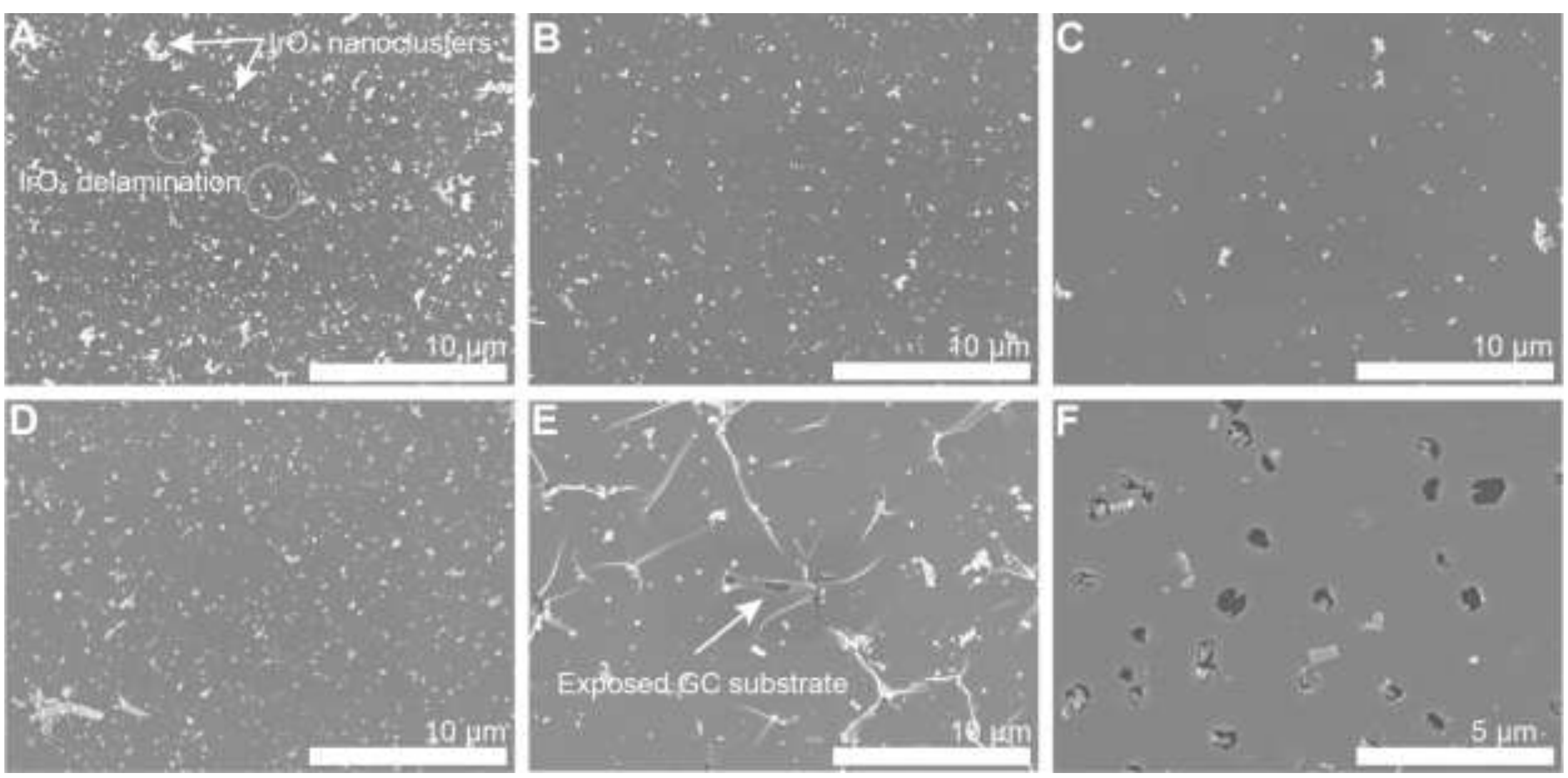

Figure S16. Postmortem SEM of spray-coated IrO_x Anodes. SEM images of postmortem anodes fabricated via **SC** at $5{\cdot}10^3$ magnification after 50 CV scans, corresponding to **(A)** uncoated anode (IrO_x|GC) where small spots of catalyst delamination from the GC substrate are observed. The coated samples fabricated via **PE-ALD** are displayed for an increasing concentration of PDMS in toluene as follows: **(B)** 2 $mg{\cdot}L^{-1}$, **(C)** 5 $mg{\cdot}L^{-1}$, **(D)** 10 $mg{\cdot}L^{-1}$, where a black area corresponds to the exposed GC substrate and some cracks are present and **(E)** 20 $mg{\cdot}L^{-1}$.**(F)** Corresponds to a considerably higher concentration (880 $mg{\cdot}L^{-1}$) and lower magnification ($10{\cdot}10^3$) where blistering effect is observed resulting from co-delamination of the catalyst-coating system (SiO_x|IrO_x) from the GC substrate.
